# Synthesis and Antiplasmodial Evaluation of 4-Carboxamido- and 4-Alkoxy-2-Trichloromethyl Quinazolines

**DOI:** 10.3390/molecules25173929

**Published:** 2020-08-27

**Authors:** Dyhia Amrane, Armand Gellis, Sébastien Hutter, Marion Prieri, Pierre Verhaeghe, Nadine Azas, Patrice Vanelle, Nicolas Primas

**Affiliations:** 1 Aix Marseille Univ, CNRS, ICR UMR 7273, Equipe Pharmaco-Chimie Radicalaire, Faculté de Pharmacie, 13385 Marseille CEDEX 05, France; dyhia.amrane@etu.univ-amu.fr (D.A.); armand.gellis@univ-amu.fr (A.G.); Marion.Prieri@newcastle.ac.uk (M.P.); 2 IHU Méditerranée Infection, UMR VITROME, IRD, SSA, Mycology & Tropical Eucaryotic Pathogens, Aix Marseille Univ, 13005 Marseille CEDEX 05, France; sebastien.hutter@univ-amu.fr (S.H.); nadine.azas@univ-amu.fr (N.A.); 3 LCC-CNRS Université de Toulouse, CNRS, UPS, 31400 Toulouse, France; pierre.verhaeghe@lcc-toulouse.fr

**Keywords:** 2-trichloromethylquinazoline, *Plasmodium falciparum*, in vitro HepG2 cytotoxicity, structure-activity relationships

## Abstract

From three previously identified antiplasmodial hit compounds (**A**–**C**) and inactive series (**D**), all based on a 2-trichloromethylquinazoline scaffold, we conducted a structure-activity relationship (SAR) study at position four of the quinazoline ring by synthesizing 42 novel derivatives bearing either a carboxamido- or an alkoxy-group, to identify antiplasmodial compounds and to enrich the knowledge about the 2-trichloromethylquinazoline antiplasmodial pharmacophore. All compounds were evaluated in vitro for their cytotoxicity towards the HepG2 cell line and their activity against the multiresistant K1 *P. falciparum* strain, using doxorubicin, chloroquine and doxycycline as reference drugs. Four hit-compounds (EC_50_ K1 *P. falciparum* ≤ 2 µM and SI ≥ 20) were identified among 4-carboxamido derivatives (**2**, **9**, **16,** and **24**) and two among 4-alkoxy derivatives (**41** and **44**). Regarding the two most potent molecules (**16** and **41**), five derivatives without a 2-CCl_3_ group were prepared, evaluated, and appeared totally inactive (EC_50_ > 50 µM), showing that the 2-trichloromethyl group was mandatory for the antiplasmodial activity.

## 1. Introduction

Among parasitic diseases, malaria remains the leading cause of death in 2020. According to the World Malaria Report 2019 [1], some 405,000 deaths were reported in 2018 for 228 million cases of malaria worldwide. Children under five years old are the most vulnerable group, accounting for 67% of the deaths. Malaria in humans is caused by five different species of *Plasmodium* protozoa, among which, *P. falciparum* is by far the most lethal, mainly in Africa. *P. vivax*, responsible for relapses, is mainly found in South-East Asia. The parasites are vectorized during blood-meal by infected female mosquitoes belonging to the *Anopheles* genus. Huge efforts have been made to control and eradicate the disease, leading to a significant reduction in the number of deaths, from 585,000 in 2010 to 405,000 in 2018 [1]. Despite this improvement, the emergence of forms of resistance to the first line treatments recommended by the WHO, the artemisinin-combination therapies (ACT), currently threaten efforts to control the disease. PfKelch13 mutations have been identified as molecular markers of artemisinin resistance [2]. Recognized since 2002–2004, artemisinin resistance was originally located mainly in the Greater Mekong Subarea [3], and failure rates for first-line ACTs were found to be as high as 93% in Thailand [1]. However, the emergence of potential artemisinin-resistance PfKelch13 mutations was reported in African regions, leading to a major concern [4]. Thus, research efforts need to be pursued with a view to discovering new chemical entities with new mechanisms of action against *Plasmodium*.

**Figure 1:**
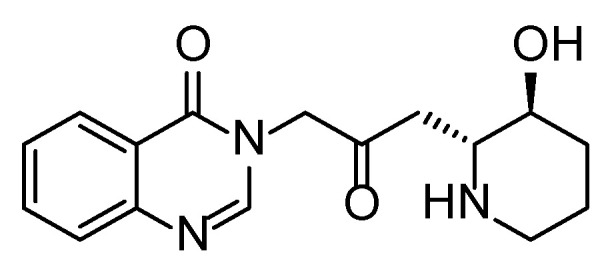
Structure of natural quinazoline-containing febrifugine.

Among small molecules displaying antiplasmodial activity, febrifugine (Figure 1), extracted from *Dichroa febrifuga*, is a natural alkaloid containing a quinazoline scaffold [5].

Our group, dedicated to the development of a new antiplasmodial [6,7,8], previously described the antiplasmodial activity of 4-aryl-2-trichloromethylquinazoline derivatives [9]. Indeed, 4-(4′-fluorophenyl)-2-trichloromethylquinazoline **A** (Table 1) showed an efficacy concentration 50% (EC_50_) of 2.5 µM against the chloroquino-resitant K1 strain of *P. falciparum* and a cytotoxicity concentration 50% (CC_50_) above 125 µM toward the human HepG2 cell line, compared with chloroquine, doxycycline and doxorubicin used as reference drugs. Indeed, chloroquine and doxycycline were still in use for antimalarial chemoprophylaxis along with the association of atovaquone and proguanil. Introducing an oxygen atom as a linker between position four of the quinazoline moiety and the phenyl group led to a two-fold improvement in antiplasmodial activity for compound **B** (Table 1) [10]. To study the Structure-Activity Relationships (SAR) related to the nature of the linker, an atom of carbon was added to provide a two-atom linker in 4-benzyloxy-2-trichloromethylquinazoline derivatives [11]. The resulting modification did not improve antiplasmodial activity, as shown with compound **C** (Table 1). When introducing a sulfonamide linker, affording 4-arylsulfonamido-2-trichloromethylquinazoline derivatives [12], the activity against *P. falciparum* was lost despite a significant decrease of the cytotoxicity (Table 1).

The aim of current study was to explore other 2-trichloromethylquinazolines bearing a two-atom linker in 4-carboxamido series, analogous to the 4-sulfonamide series **D**, and in 4-alkoxy series analogous to compounds **B** or **C** in aryloxy and benzyloxy series, respectively. The syntheses and in vitro biological evaluations are presented and discussed.

## 2. Results

### 2.1. Synthesis

#### 2.1.1. Synthesis of 4-Carboxamido-2-Trichloromethylquinazoline Series

We first sought to obtain the target compounds using key intermediate 4-amino-2-trichloromethylquinazoline (**1**), previously described as generating the sulfonamide derivatives belonging to the **D** series [12]. Thus, using 4-chlorobenzoyl chloride in presence of sodium hydride in DMF, we obtained a mixture of starting material (**1**) and the formation of dibenzamide (**3**) resulting from a double substitution of the amino group (Table 2, Entry 1). Surprisingly, no formation of target compound (**2**) was observed. The same conditions were used at 0 °C and did not afford (**2**) (Entry 2). Increasing both base and acyl chloride only provided a mixture of (**1**) and dibenzamide (**3**) (Entries 3–4). Neither switching solvent types from DMF to THF (Entry 5), nor switching base types from NaH to *t*BuOK (Entry 6) yielded (**2**). The use of organic bases such as NaHMDS or NEt_3_ was also unsuccessful, in particular with NEt_3_ with no conversion observed (Entries 7–9). Finally, in order to isolate the dibenzamide (**3**), the use of 5 equiv. of NaH led to total conversion and led to (**3**) with 90% yield (Entry 10).

**Scheme 1:**
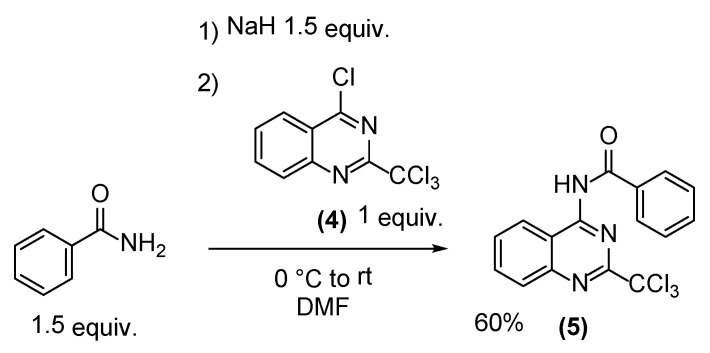
Preparation of benzamide (**5**) from chlorimine (**4**).

In view of the ineffectiveness of this synthetic route, we changed tactics and started from 4-chloro-2-trichloromethylquinazoline (**4**), previously described [13] and used by our team in S_N_Ar reactions with various nucleophilic reagents [10,14,15] or Suzuki-Miyaura cross-coupling reactions [9]. Thus, commercial benzamide was deprotonated using NaH in DMF and reacted with chlorimine (**4**) in DMF, leading to the target compound (**5**) in 60% yield (Scheme 1). As the conversion of the reaction was total and the yield was only impaired by the purification step, the reaction conditions were not modified and various commercially available substituted benzamides, heteroarylcarboxamides, and alkylcarboxamides were reacted (Table 3). The benzamides did not show a clear relationship between yields obtained (from 54 to 98%) and their electron-donating/-withdrawing behavior, nor between yields and substituents position borne by their phenyl ring (**3**, **5**–**16**). For pyridine-containing carboxamides (**17**–**19**), poor to good yields were obtained (22–81%) related to purification issues in silica gel chromatography, even though the silica was deactivated by NEt_3_. With alkylcarboxamides, yields were generally lower than with benzamides (31–55%) (**20**–**25**).

#### 2.1.2. Synthesis of 4-Alkoxy-2-Trichloromethylquinazoline Series

**Scheme 2:**
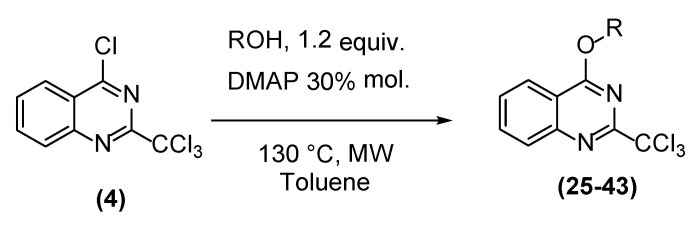
Preparation of 4-alkoxy-2-trichloromethylquinazoline (**25**–**43**) from chlorimine (**4**).

As we previously showed that the introduction of an oxygen-containing substituents at position 4 of the quinazoline scaffold was favorable for the activity (compounds **B** and **C**, Table 1), we looked for oxygen-containing substituent in aliphatic series rather than previous aryl-containing derivatives. Using a previously reported reaction, a DMAP-catalyzed S_N_Ar reaction between (**4**) and various aliphatic alcohols was conducted (Scheme 2). First, the reaction was conducted with linear aliphatic alcohol from C_1_ to C_4_ and afforded corresponding alkoxy derivatives (**26**–**29**) in good yields (75–81%) (Table 4). Isopropoxy derivative (**30**) was obtained with the modest yield of 53%. Propargyl alcohols led to target compounds (**31**–**33**) in very good yields (87–90%). Reaction with ethylene glycol led to (**34**) with a modest yield (42%) due to dimer formation, whereas 2-methoxyethanol afforded (**35**) in nearly quantitative yield. Halogen alkyl alcohols yielded corresponding products (**36**–**40**) in good yields (70–79%) except for (**38**), which was obtained as an oil (28%). Finally, various substituted aminoalcohols yielded target compounds (**41**–**44**) in modest (**44**, 20%) to good yields (**42**, 79%).

### 2.2. Biological Evaluations

All synthesized molecules were then evaluated in vitro against the multi-resistant K1 *P. falciparum* strain, by determining their 50% efficacy concentration (EC_50_), and compared with two antimalarial drug-compounds: chloroquine and doxycycline. In parallel, these molecules were assessed in vitro on the HepG2 human hepatocyte cell line, by determining their 50% cytotoxic concentrations (CC_50_) and comparing them to that of doxorubicin, used as a cytotoxic reference drug-compound, in order to calculate their respective selectivity indices (SI = CC_50_/EC_50_). The results are presented in Table 3 and Table 4.

### 2.3. Structure-Activity Relationships (SAR)

#### 2.3.1. SAR of 4-Carboxamido-2-Trichloromethylquinazoline Series

For some synthesized compounds, lack of solubility hampered full determination of cytotoxicity (**8**, **11**, **17**, **20**–**21**, and **24**) but not evaluation of antiplasmodial activity. It should be noted that in series **D**, there was no issue concerning solubility of sulfonamide derivatives [12]. For sufficiently soluble derivatives, cytotoxicity values ranged from 16.4 to 72.9 µM, high compared to that of doxorubicin (0.2 µM). Interestingly, contrary to the sulfonamides in series **D**, the carboxamide analogs exhibited an antiplasmodial activity (0.94–14.5 µM), including dibenzamide (**3**) (1.46 µM). Unsubstituted benzamide (**5**) displayed a moderate activity of 1.76 µM. The introduction of a strong electron-withdrawing group such as nitro group reduced the activity, and ortho (**8**) substitution was better than meta (**7**) and para (**6**). The influence of the substitution was inverted with a fluorine atom such as para (**9**) and displayed better activity (1.34 µM) than meta (**10**) and para substitution (**11**). The same profile was observed with chloro-substituted benzamides leading to a micromolar antiplasmodial activity for para-Cl (**2**). Substituting para-Cl for para-Br (**14**) was not in favor of the activity (1.30 µM vs. 0.99µM). Introducing para-CN (**15**) was also more detrimental to the activity (3.5 µM), while para-OMe (**16**) showed the best activity against K1 *P. falciparum* (0.94 µM, SI = 29.5). The pyridine-containing carboxamides (**17**–**18**) were less active (3.90–14.5 µM), except for isonicotinamide (**19**), which had an EC_50_ of 1.8 µM. Regarding the alkylcarboxamides, acetamide (**20**) was not active (>10 µM). The addition of carbon atoms to the alkyl chain led to more cluttered analogs that displayed similar activity to benzamides, like analogs (**23**) or (**24**) (1.8 and 1.6 µM). Cyclohexylcarboxamide (**25**) activity was slightly lower than that of terbutylcarboxamide (**23**). In conclusion, to provide the best antiplasmodial activity with good SI, para-substituted benzamides with chlorine, fluorine, methoxy, or non-cyclic alkylcarboxamide with fatty carbon chain were required.

#### 2.3.2. SAR of 4-Alkoxy-2-Trichloromethylquinazoline Series

For the whole series, the solubility of the compounds was acceptable. Apart from alcohol (**34**) with a cytotoxicity value of 7.9 µM, the rest of the series was not cytotoxic (23.4–101.5 µM), in comparison with doxorubicin. In this alkoxy series, amino-containing side chain were the most potent derivatives. We previously showed that 2-(trichloromethyl)quinazolin-4-ol (R = H) was not active against W2 *P. falciparum* [10]. Interestingly, when R = Me (**26**), a moderate activity was observed (EC_50_ = 7.1 µM), increasing with the number of carbons in the alkyl chain, up to 3 carbon atoms. The best activity (3.5 µM) was obtained with a propoxy substituent (**28**), whereas with butoxy (**29**) the activity decreased again (5.8 µM), the compound even becoming inactive with the bulkier isopropoxy group (**30**). Compound (**31**) resulting from the reaction of propargyl alcohol showed a modest activity, which was lost with substituted propargyl alcohols (**32**–**33**). Compound (**34**) bearing a 2-hydroxyethoxy group was almost inactive (10 µM). Replacing the hydroxyl group of (**34**) by a methoxy group (**35**) or a chlorine atom (**36**) afforded more active derivatives (2.3 and 2.2 µM). The addition of one (**37**) or two atoms of carbon (**38**) in the chain of (**36**) decreased the activity two-fold (4.2 and 4.3 µM). The replacement of the chlorine atom of (**36**) by a fluorine atom (**39**) led to a decrease in activity (8.0 µM) while a bromine atom (**40**) preserved it. Among aminoethoxy derivatives (**41**–**44**), diethylamino (**41**) was the most potent, with a submicromolar antiplasmodial activity (0.9 µM) and a SI of 35.9. Cyclic analogs (**43**) and (**44**) were slightly less active than (**41**) whereas acetamide (**42**) was clearly less potent. In conclusion, we succeed in obtaining 4-*O*-substituted-quinazoline from inactive derivative 4-alcohol [10] to submicromolar active compounds in 4-alkoxyamino series.

**Figure 2:**
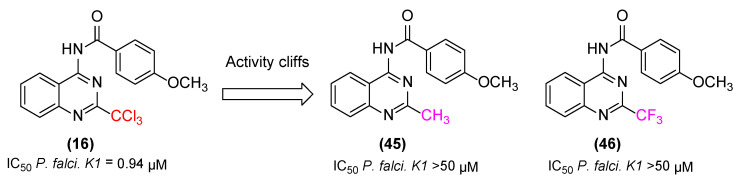
Comparison of the in vitro antiplasmodial activities of hit molecule (**16**) and negative controls (**45**–**46**).

**Figure 3:**
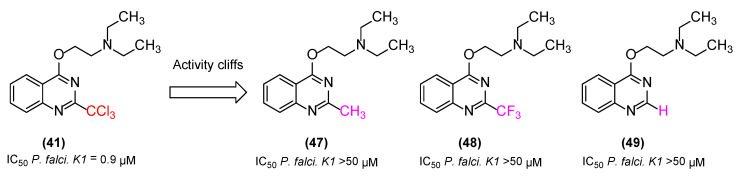
Comparison of the in vitro antiplasmodial activities of hit molecule (**41**) and negative controls (**47**–**49**).

To confirm the key role played by the 2-trichloromethylquinazoline moiety of the most potent compounds (**16**) and (**41**) in each studied series, derivatives without a -CCl_3_ group were synthesized for the two hit molecules: dehalogenated analogs (**45**, **47**), 2-trifluromethyl analogs (**46**, **48**) and unsubstituted analog (**49**) (Figure 2 and Figure 3). All molecules were inactive against *P. falciparum,* illustrating the key role played by the -CCl_3_ group and consistent with our previous results. Thus, activity cliffs (ACs) [16] were observed when the -CCl_3_ group was replaced by similar group (-CF_3_, CH_3_) or atom (H) as a 50-fold drop of antiplasmodial activity.

The mechanism of action of the 2-trichloromethylquinazolines is not known, but it was clearly related to the presence of the -CCl_3_ group, which could act as a possibly alkylating group as methyl and trifluoromethyl analogues were inactive or the -CCl_3_ group could generate sigma-holes [17] with sulphur nucleophiles in cysteine proteases such as Falcipains as potential plasmodial targets [18].

## 3. Material and Methods

### 3.1. General

Melting points were determined on a Köfler melting point apparatus (Wagner & Munz GmbH, München, Germany) and are uncorrected. Elemental analyses were carried out at the Spectropole, Faculté des Sciences de Saint-Jêrome (Marseille) with a Thermo Finnigan EA1112 analyzer (Thermo Finnigan, San Jose, CA, USA). NMR spectra were recorded on a Bruker AV (Billerica, MA, USA) 200 or AV 250 spectrometers or a Bruker Avance NEO 400MHz NanoBay spectrometer at the Faculté de Pharmacie of Marseille or on a Bruker Avance III nanobay 400 MHz spectrometer at the Spectropole, Faculté des Sciences de Saint-Jêrome (Marseille). (^1^H NMR: reference CHCl_3_ δ = 7.26 ppm, reference DMSO-*d*_6_ δ = 2.50 ppm and ^13^C NMR: reference CHCl_3_ δ = 76.9 ppm, reference DMSO-*d*_6_ δ = 39.52 ppm). The following adsorbent was used for column chromatography: silica gel 60 (Merck KGaA, Darmstadt, Germany, particle size 0.063–0.200 mm, 70–230 mesh ASTM). TLC was performed on 5 cm × 10 cm aluminum plates coated with silica gel 60F-254 (Merck) in an appropriate eluent. Visualization was performed with ultraviolet light (234 nm). Purity of synthesized compounds was checked by LC/MS analyses, which were realized at the Faculté de Pharmacie of Marseille with a Thermo Scientific Accela High Speed LC System^®^ (Waltham, MA, USA) coupled using a single quadrupole mass spectrometer Thermo MSQ Plus^®^. The RP-HPLC column is a Thermo Hypersil Gold^®^ 50 × 2.1 mm (C_18_ bounded), with particles of a diameter of 1.9 mm. The volume of sample injected on the column is 1 µL. Chromatographic analysis, total duration of 8 min, is on the gradient of the following solvents: t = 0 min, methanol/water 50:50; 0 < t < 4 min, linear increase in the proportion of methanol to a methanol/water ratio of 95:5; 4 < t < 6 min, methanol/water 95:5; 6 < t < 7 min, linear decrease in the proportion of methanol to return to a methanol/water ratio of 50:50; 6 < t < 7 min, methanol/water 50:50. The water used was buffered with ammonium acetate 5 mM. The flow rate of the mobile phase was 0.3 mL/min. The retention times (*t_R_*) of the molecules analyzed are indicated in min. The microwave reactions were performed using multimode reactors: ETHOS Synth Lab station and MicroSYNTH^®^ Lab terminal 1024 (Ethos start, MLS GmbH, Leutkirch, Germany.); or monomode reactors: Biotage Initiator^®^ classic in sealed vials with a power output of 0 to 400 W. 4-Chloro-2-methylquinazoline, 4-chloro-2-trifluoromethylquinazoline, and 4-chloroquinazoline were purchased from Sigma-Aldrich (Saint Louis, MO, USA) or Fluorochem (Derbyshire, UK). The following Supplementary Materials are available online: 1H-NMR, 13C-NMR and HRMS data spectra of compounds 2, 9, 16, 24, 41, 44, 45, 46, 48 and 49.

### 3.2. 4-Amino-2-Trichloromethylquinazoline (**1**)

In a microwave vial equipped with a magnetic stir bar, 4-chloro-2-trichloromethylquinazoline [13] (700 mg, 2.48 mmol, 1 equiv.) and ammonia solution in THF (0.4 M, 18.6 mL, 7.45 mmol, 3 equiv.). The vial was capped and the suspension was then heated at 140 °C for 15 min (8 bar). The volatiles were removed under vacuum. The residue was poured into EtOAc (100 mL) and extracted twice with brine (30 mL). The organic layer was dried with Na_2_SO_4_, filtered and evaporated to afford (**1**) as white solid (650 mg, 100%). Mp 199 °C. ^1^H-NMR (DMSO-*d*_6_): δ 8.33–8.27 (m, 3H), 7.90–7.77 (m, 2H), 7.65–7.57 (m, 1H). ^13^C-NMR (DMSO-*d*_6_): δ 163.2, 160.9, 148.8, 134.1, 128.3, 127.5, 123.9, 113.4, 98.4. LC-MS (ESI) Tr 2.37 min, *m*/*z* [M + H]^+^ Calcd: 261.96, Found: 262.03. Anal. calcd. for C_9_H_6_Cl_3_N_3_: C, 41.18; H, 2.30; N, 16.01. Found: C, 41.43; H, 2.31; N, 16.29.

### 3.3. 4-Chloro-N-(4-Chlorobenzoyl)-N-(2-Trichloromethylquinazolin-4-yl)benzamide (**3**)

To a solution of 60% sodium hydride in oil (152 mg, 3.81 mmol, 5.0 equiv.) in dry THF (4 mL) at 0 °C was slowly added 4-amino-2-trichloromethylquinazoline (200 mg, 0.76 mmol, 1.0 equiv). After 30 min of stirring at rt, the reaction mixture was cooled again and the 4-chlorocarboxamide (1.52 mmol, 2.0 equiv.) was added portion wise. After this addition, the reaction was stirred at rt until the starting material disappeared. The excess of NaH was hydrolyzed with ice at 0 °C. The mixture was extracted with EtOAc and washed three times with brine. The organic layer was dried with Na_2_SO_4_, filtered, and evaporated. The crude residue was triturated in dichloromethane, filtered, and recrystallized from appropriate solvent to give the desired compound.

Yield 90%. White solid. Mp 218.2 °C, (isopropanol). ^1^H NMR (400 MHz, DMSO-*d*_6_) δ = 8.33–8.23 (m, 2H), 8.20 (d, *J* = 8.2 Hz, 1H), 8.03 (ddd, *J* = 8.3, 6.3, 1.8 Hz, 1H), 7.95–7.90 (m, 4H), 7.61–7.55 (m, 4H). ^13^C NMR (101 MHz, DMSO-*d*_6_) δ *=* 172.0 (2C), 169.6, 161.9, 158.7, 150.5, 141.4, 138.7, 137.0, 131.7, 131.5, 131.1 (4C), 129.5, 129.4 (4C), 124.9, 117.9, 95.6. LC-MS (ESI+) *t_R_* 4.95 min, *m*/*z* [M + H]^+^ 539.83/541.81/542.78/544.72/547.72. MW: 539.63 g.mol^−1^. HRMS *m*/*z* [M + H]^+^ calcd for C_23_H_12_Cl_5_N_3_O_2_: 539.9418, found: 539.9417.

### 3.4. 4-Chloro-2-Trichloromethylquinazoline (**4**)

4-Chloro-2-trichloromethylquinazoline (**1**) was prepared as described in the literature using a multimode microwave reactor at 800 W [13]. Yield 82%. White solid. Mp 127 °C (lit. 127 °C) [13]. ^1^H NMR (CDCl_3_, 200 MHz) δ = 7.82–7.90 (m, 1H), 8.03–8.12 (m, 1H), 8.20–8.24 (m, 1H), 8.33–8.38 (m, 1H).

### 3.5. General Procedure for the Preparation of Compounds (**2**), (**5**–**25**)

To a solution of the appropriate carboxamide compound (1.06 mmol, 1.5 equiv.) in dry DMF (3 mL) at 0 °C under N_2_, 60% sodium hydride in oil (25.5 mg, 1.06 mmol, 1.5 equiv) were added portion wise. The resulting mixture were added dropwise to a solution of 4-chloro-2-(trichloromethyl)quinazoline (**4**) (200 mg, 0.71 mmol, 1.0 equiv.) in dry DMF (2 mL) at 0 °C under N_2_. The reaction was stirred overnight at rt. Then, the excess of NaH was hydrolyzed with ice. The reaction mixture was extracted with EtOAc and washed three times with brine. The organic layer was dried with Na_2_SO_4_, filtered, and evaporated. The crude product was purified by silica gel column chromatography and recrystallized from appropriate solvent to give the desired compound.

#### 3.5.1. 4-Chloro-N-(2-Trichloromethylquinazolin-4-yl)benzamide (**2**)

Yield 74%. White solid. Mp 199 °C, (isopropanol). ^1^H NMR (400 MHz, DMSO-*d*_6_) δ = 11.74 (s, 1H), 8.32 (d, *J* = 8.4 Hz, 1H), 8.18–8.10 (m, 2H), 8.00 (d, *J* = 8.5 Hz, 2H), 7.90–7.80 (m, 1H), 7.62 (d, *J* = 8.5 Hz, 2H). ^13^C NMR (101 MHz, DMSO-*d*_6_) δ = 167.3, 160.4, 159.4, 150.1, 137.3, 135.7, 132.4, 130.6 (2C), 129.3, 128.6 (3C), 125.9, 117.2, 96.8. LC-MS (ESI+) *t_R_* 3.92 min, *m*/*z* [M + H] ^+^ 399.03/401.59/403.98/405.94 MW: 367.62 g.mol^−1^. HRMS *m*/*z* [M + H]^+^ calcd for C_16_H_9_Cl_4_N_3_: 401.9544, Found: 401.9545.

#### 3.5.2. N-(2-Trichloromethylquinazolin-4-yl)benzamide (**5**)

Yield 60%. White solid. Mp 183 °C, (isopropanol). ^1^H NMR (400 MHz, DMSO-*d*_6_) δ = 11.67 (s, 1H), 8.30 (d, *J* = 8.3 Hz, 1H), 8.15–8.09 (m, 2H), 8.02–8.00 (m, 2H), 7.86–7.82 (m, 1H), 7.65 (t, *J* = 7.4 Hz, 1H), 7.55 (t, *J* = 7.6 Hz, 2H). ^13^C NMR (101 MHz, DMSO-*d*_6_) δ = 168.1, 160.6, 159.5, 150.1, 135.6, 133.4, 132.5, 129.3, 128.6 (2C), 128.5, 128.5 (2C), 125.9, 117.3, 96.8. LC-MS (ESI+) *t_R_* 3.38 min, *m*/*z* [M + H]^+^ 365.81/367.02/369.83. MW: 366.63 g.mol^−1^. HRMS *m*/*z* [M + H]^+^ calcd for C_16_H_10_Cl_3_N_3_O: 365.9962, Found: 365.9961.

#### 3.5.3. 4-Nitro-N-(2-Trichloromethylquinazolin-4-yl)benzamide (**6**)

Yield 67%. Yellow solid. Mp 214 °C, (isopropanol). ^1^H NMR (400 MHz, DMSO-*d*_6_) δ = 12.02 (s, 1H), 8.42 (d, *J* = 8.3 Hz, 1H), 8.36–8.33 (m, 2H), 8.14 (dd, *J* = 6.3 Hz, 2.3 Hz, 4H), 7.92–7.85 (m, 1H). ^13^C NMR (101 MHz, DMSO-*d*_6_) δ = 167.4, 159.8, 159.2, 150.0, 149.4, 139.7, 135.8, 129.9 (2C), 129.4, 128.1, 125.7, 123.6 (2C), 116.7, 96.6. LC-MS (ESI+) *t_R_* 3.55 min, *m*/*z* [M + H]^+^ 410.98/413.01. MW: 411.63 g.mol^−1^. HRMS *m*/*z* [M + H]^+^ calcd for C_16_H_9_Cl_3_N_4_O_3_: 410.9813, Found: 410.9815.

#### 3.5.4. 3-Nitro-N-(2-Trichloromethylquinazolin-4-yl)benzamide (**7**)

Yield 60%. White solid. Mp 196 °C, (isopropanol). ^1^H NMR (400 MHz, DMSO-*d*_6_) δ = 12.02 (s, 1H), 8.79 (s, 1H), 8.48 (d, *J* = 8.2 Hz, 1H), 8.43–8.36 (m, 2H), 8.19–8.12 (m, 2H), 7.93–7.79 (m, 2H). ^13^C NMR (101 MHz, DMSO-*d*_6_) δ = 166.4, 160.0, 159.3, 150.1, 147.7, 135.8, 135.2, 134.9, 130.3, 129.4, 128.6, 126.8, 126.0, 123.3, 117.0, 96.7. LC-MS (ESI+) *t_R_* 3.50 min, *m*/*z* [M + H]^+^ 410.98/412.97/410.98. MW: 411.63 g.mol^−1^. HRMS *m*/*z* [M+Na]^+^ calcd for C_16_H_9_Cl_3_N_4_O_3_: 432.9632, Found: 432.9627.

#### 3.5.5. 2-Nitro-N-(2-Trichloromethylquinazolin-4-yl)benzamide (**8**)

Yield 54%. White solid. Mp 257 °C, (isopropanol). ^1^H NMR (400 MHz, DMSO-*d*_6_) δ = 12.19 (s, 1H), 8.71 (d, *J* = 8.1 Hz, 1H), 8.33 (dd, *J* = 8.3, 0.8 Hz, 1H), 8.17–8.07 (m, 1H), 8.04 (d, *J* = 7.6 Hz, 1H), 7.90 (ddd, *J* = 8.2 Hz, 7.0 Hz, 1.2 Hz, 1H), 7.79 (td, *J* = 7.5 Hz, 1.1 Hz, 1H), 7.73–7.66 (m, 1H), 7.63 (dd, *J* = 7.5 Hz, 1.2 Hz, 1H). ^13^C NMR (101 MHz, DMSO-*d*_6_) δ = 168.0, 158.7, 157.4, 149.3, 144.9, 135.6, 135.1, 134.1, 130.1, 129.5, 128.7, 127.1, 124.9, 124.4, 114.5, 96.2. LC-MS (ESI+) *t_R_* 3.23 min, *m*/*z* [M + H]^+^ 410.99/412.90/414.88. MW: 411.63 g.mol^−1^. HRMS *m*/*z* [M + H]^+^ calcd for C_16_H_9_Cl_3_N_4_O_3_: 410.9813, Found: 410.9804.

#### 3.5.6. 4-Fluoro-N-(2-Trichloromethylquinazolin-4-yl)benzamide (**9**)

Yield 88%. White solid. Mp 166 °C, (isopropanol). ^1^H NMR (400 MHz, DMSO-*d*_6_) δ = 11.69 (s, 1H), 8.30 (d, *J* = 8.4 Hz, 1H), 8.17–8.11 (m, 2H), 8.11–8.06 (m, 2H), 7.89–7.81 (m, 1H), 7.39 (t, *J* = 8.8 Hz, 2H). ^13^C NMR (101 MHz, DMSO-*d*_6_) δ 167.0, 164.6 (d, *J* = 250.5 Hz), 160.5, 159.4, 150.0, 135.6, 131.5 (d, *J* = 9.3 Hz), 129.9 (d, *J* = 2.8 Hz), 129.2, 128.5, 125.9, 117.2, 115.5 (d, *J* = 22.0 Hz), 96.8. LC-MS (ESI+) *t_R_* 3.58 min, *m*/*z* [M + H]^+^ 384.05/386.02/387.95. MW: 384.62 g.mol^−1^. HRMS *m*/*z* [M + H]^+^ calcd for C_16_H_9_Cl_3_FN_3_O: 405.9687, Found: 405.9688.

#### 3.5.7. 3-Fluoro-N-(2-Trichloromethylquinazolin-4-yl)benzamide (**10**)

Yield 80%. Yellow solid. Mp 152 °C, (isopropanol). ^1^H NMR (400 MHz, DMSO-*d*_6_) δ = 11.74 (s, 1H), 8.32 (d, *J* = 6.5 Hz, 1H), 8.17–8.09 (m, 2H), 7.89–7.78 (m, 3H), 7.63–7.57 (m, 1H), 7.50 (dt, *J* = 8.6, 2.1 Hz, 1H). ^13^C NMR (101 MHz, DMSO-*d*_6_) δ = 166.9 (d, *J* = 2.6 Hz), 161.9 (d, *J* = 244.5 Hz), 160.2, 159.4, 150.1, 135.8 (d, *J* = 7.2 Hz), 135.7, 130.6 (d, *J* = 8.0 Hz), 129.3, 128.6, 125.9, 124.7 (d, *J* = 2.7 Hz), 119.3 (d, *J* = 21.2 Hz), 117.2, 115.4 (d, *J* = 23.2 Hz), 96.7. LC-MS (ESI+) *t_R_* 3.57 min, *m*/*z* [M + H]^+^ 383.99/385.06/386.08/388.05/389.05. MW: 384.62 g.mol^−1^. HRMS m/z [M + Na]^+^ calcd for C_16_H_9_Cl_3_FN_3_O: 405.9687, Found: 405.9688.

#### 3.5.8. 2-Fluoro-N-(2-Trichloromethylquinazolin-4-yl)benzamide (**11**)

Yield 98%. White solid. Mp 190 °C, (isopropanol). ^1^H NMR (400 MHz, DMSO-*d*_6_) δ = 11.95 (s, 1H), 8.53 (d, *J* = 8.4 Hz, 1H), 8.11 (q, *J* = 8.5 Hz, 2H), 7.93–7.86 (m, 1H), 7.71 (td, *J* = 7.5, 1.2 Hz, 1H), 7.57–7.50 (m, 1H), 7.30 (t, *J* = 7.5 Hz, 1H), 7.21 (dd, *J* = 10.3, 8.8 Hz, 1H). ^13^C NMR (101 MHz, DMSO-*d*_6_) δ = 166.1, 159.1, 159.0, 158.8 (d, *J* = 249.4 Hz), 149.8, 135.6, 133.0 (d, *J* = 8.7 Hz), 130.2 (d, *J* = 2.5 Hz), 129.5, 128.6, 124.8, 124.7 (d, *J* = 3.4 Hz), 124.1 (d, *J* = 13.9 Hz), 116.1 (d, *J* = 21.7 Hz), 115.6, 96.5. LC-MS (ESI+) *t_R_* 3.44 min, *m*/*z* [M + H] ^+^384.01/386.00/388.00. MW: 384.62 g.mol^−1^. HRMS *m*/*z* [M + H] ^+^ calcd for C_16_H_9_Cl_3_FN_3_O: 383.9868, Found: 382.9795.

#### 3.5.9. 3-Chloro-N-(2-Trichloromethylquinazolin-4-yl)benzamide (**12**)

Yield 82%. White solid. Mp 139 °C, (isopropanol). ^1^H NMR (400 MHz, DMSO-*d*_6_) δ = 11.81 (s, 1H), 8.35 (d, *J* = 8.4 Hz, 1H), 8.19–8.10 (m, 2H), 8.06 (s, 1H), 7.94 (d, *J* = 8.3 Hz, 1H), 7.90–7.82 (m, 1H), 7.71 (d, *J* = 8.2 Hz, 1H), 7.58 (t, *J* = 7.9 Hz, 1H). ^13^C NMR (101 MHz, DMSO-*d*_6_) δ = 166.8, 160.2, 159.4, 150.1, 135.7, 135.6, 133.2, 132.2, 130.4, 129.3, 128.6, 128.3, 127.3, 126.0, 117.1, 96.8. LC-MS (ESI+) *t_R_* 3.98 min, *m*/*z* [M + H]^+^ 401.92/403.97/405.89. MW: 401.07 g.mol^−1^. HRMS *m*/*z* [M + H]^+^ calcd for C_16_H_9_Cl_4_N_3_O: 401.9544, Found: 401.9546.

#### 3.5.10. 2-Chloro-N-(2-Trichloromethylquinazolin-4-yl)benzamide (**13**)

Yield 83%. Yellow solid. Mp 171 °C, (isopropanol). ^1^H NMR (400 MHz, DMSO-*d*_6_) δ = 12.02 (s, 1H), 8.59 (d, *J* = 8.4 Hz, 1H), 8.11 (dd, *J* = 14.1, 7.3 Hz, 2H), 7.93–7.86 (m, 1H), 7.58 (dd, *J* = 7.3, 1.6 Hz, 1H), 7.51–7.34 (m, 3H). ^13^C NMR (101 MHz, DMSO-*d*_6_) 168.2, 159.1, 158.4, 149.7, 136.1, 135.6, 131.2, 129.79, 129.78, 129.5, 129.1, 128.6, 127.4, 124.7, 115.3, 96.4. LC-MS (ESI+) *t_R_* 3.66 min, *m*/*z* [M + H]^+^ 398.83/401.88/403.98. MW: 401.07 g.mol^−1^. HRMS *m*/*z* [M + H]^+^ calcd for C_16_H_9_Cl_4_N_3_O: 401.9544, Found: 401.9543.

#### 3.5.11. 4-Bromo-N-(2-Trichloromethylquinazolin-4-yl)benzamide (**14**)

Yield 62%. Beige solid. Mp 201 °C, (isopropanol). ^1^H NMR (400 MHz, DMSO-*d*_6_) δ = 11.73 (s, 1H), 8.29 (dd, *J* = 18.3 Hz, 8.5 Hz, 1H), 8.18–8.09 (m, 2H), 7.92 (d, *J* = 8.5 Hz, 2H), 7.85 (ddd, *J* = 8.3 Hz, 5.6 Hz, 2.6 Hz, 1H), 7.76 (d, *J* = 8.5 Hz, 2H). ^13^C NMR (101 MHz, DMSO-*d*_6_) δ = 167.4, 160.3, 159.3, 150.0, 135.6, 132.7, 131.5 (2 C), 130.6 (2 C), 129.3, 128.5, 126.2, 125.8, 117.1, 96.8. LC-MS (ESI+) *t_R_* 4.20 min, *m*/*z* [M + H]^+^ 443.79/445.83/447.73/449.59. MW: 445.53 g.mol^−1^. HRMS *m*/*z* [M + H]^+^ calcd for C_16_H_9_BrCl_3_N_3_O: 445.9043, Found: 445.9046.

#### 3.5.12. 4-Cyano-N-(2-Trichloromethylquinazolin-4-yl)benzamide (**15**)

Yield 95%. White solid. Mp 170 °C, (isopropanol). ^1^H NMR (400 MHz, DMSO-*d*_6_) δ = 11.93 (s, 1H), 8.39 (d, *J* = 8.4 Hz, 1H), 8.14 (d, *J* = 3.8 Hz, 2H), 8.07 (d, *J* = 8.3 Hz, 2H), 8.01 (d, *J* = 8.3 Hz, 2H), 7.87 (dt, *J* = 8.3, 4.0 Hz, 1H). ^13^C NMR (101 MHz, DMSO-*d*_6_) δ = 167.6, 159.9, 159.2, 150.0, 138.0, 135.8, 132.5 (2 C), 129.4, 129.2 (2 C), 128.6, 125.7, 118.2, 116.8, 114.3, 96.7. LC-MS (ESI+) *t_R_* 3.03 min, *m*/*z* [M + H]^+^ 390.98/393.05/394.94. MW: 391.64 g.mol^−1^. HRMS *m*/*z* [M + H]^+^ calcd for C_17_H_9_Cl_3_N_4_O: 390.9915, Found: 390.9911.

#### 3.5.13. 4-Methoxy-N-(2-Trichloromethylquinazolin-4-yl)benzamide (**16**)

Yield 75%. Yellow solid. Mp 192 °C, (isopropanol). ^1^H NMR (400 MHz, DMSO-*d*_6_) δ = 11.52 (s, 1H), 7.40 (d, *J* = 8.4 Hz, 1H), 7.33–7.25 (m, 2H), 7.20 (d, *J* = 8.8 Hz, 2H), 7.04–6.94 (m, 1H), 6.26 (d, *J* = 8.8 Hz, 2H), 3.03 (s, 3H). ^13^C NMR (101 MHz, DMSO-*d*_6_) δ = 167.1, 162.8, 160.9, 159.5, 150.0, 135.5, 130.9 (2C), 129.1, 128.5, 126.1, 125.3, 117.45, 113.7 (2 C), 97.0, 55.5. LC-MS (ESI+) *t_R_* 3.46 min, *m*/*z* [M + H]^+^ 396.08/398.87/400.53. MW: 396.66 g.mol^−1^. HRMS *m*/*z* [M + H]^+^ calcd for C_17_H_12_Cl_3_N_3_O_2_: 396.0068, Found: 396.0062.

#### 3.5.14. N-(2-Trichloromethylquinazolin-4-yl)picolinamide (**17**)

Yield 81%. Beige solid. Mp 214 °C, (isopropanol). ^1^H NMR (400 MHz, DMSO-*d*_6_) δ = 11.72 (s, 1H), 8.76 (d, *J* = 4.5 Hz, 1H), 8.37 (d, *J* = 8.3 Hz, 1H), 8.22–8.10 (m, 4H), 7.95–7.88 (m, 1H), 7.78–7.69 (m, 1H). ^13^C NMR (101 MHz, DMSO-*d*_6_) δ = 164.0, 159.5, 159.1, 150.0, 149.1, 148.7, 138.4, 135.8, 129.5, 128.6, 127.6, 125.1, 123.1, 116.9, 96.9. LC-MS (ESI+) *t_R_* 3.4 min, *m*/*z* [M + H]^+^ 365.74/367.13/368.70/371.04. MW: 367.62 g.mol^−1^. HRMS *m*/*z* [M + H]^+^ calcd for C_15_H_9_Cl_3_N_4_O: 366.9915, Found: 366.9912.

#### 3.5.15. N-(2-Trichloromethylquinazolin-4-yl)nicotinamide (**18**)

Yield 20%. White solid. Mp 208 °C, (isopropanol). ^1^H NMR (400 MHz, DMSO-*d*_6_) δ = 11.91 (m, 1H), 9.10 (ls, 1H), 8.79 (ls, lH), 8.39 (d, *J* = 8.4 Hz, 1H), 8.30 (d, *J* = 7.9 Hz, 1H), 8.14 (d, *J* = 3.7 Hz, 2H), 7.92–7.82 (m, 1H), 7.58 (dd, *J* = 7.8, 4.8 Hz, 1H). ^13^C NMR (101 MHz, DMSO-*d*_6_) δ = 167.2, 160.1, 159.3, 152.7, 150.1, 149.4, 136.3, 135.8, 129.7, 129.4, 128.6, 125.9, 123.6, 116.9, 96.7. LC-MS (ESI+) *t_R_* 2.44 min, *m*/*z* [M + H]^+^ 365.65/368.50/371.07. MW: 367.62 g.mol^−1^. HRMS *m*/*z* [M + H]^+^ calcd for C_15_H_9_Cl_3_N_4_O: 366.9915, Found: 366.9911.

#### 3.5.16. N-(2-Trichloromethylquinazolin-4-yl)isonicotinamide (**19**)

Yield 22%. White solid. Mp 151 °C, (isopropanol). ^1^H NMR (400 MHz, DMSO-*d*_6_) δ = 12.04 (s, 1H), 8.77 (d, *J* = 5.6 Hz, 1H), 8.44 (d, *J* = 8.3 Hz, 1H), 8.15 (d, *J* = 3.6 Hz, 2H), 7.94–7.85 (m, 2H), 7.82 (d, *J* = 4.9 Hz, 2H). ^13^C NMR (101 MHz, DMSO-*d*_6_) δ = 167.7, 159.7, 159.2, 150.3, 150.0 (2C), 141.3, 135.9, 129.5, 128.6, 125.7, 122.0 (2C), 116.7, 96.7. LC-MS (ESI+) *t_R_* 2.38 min, *m*/*z* [M + H]^+^ 365.72/368.60/371.04. MW: 367.62 g.mol^−1^. HRMS *m*/*z* [M + H]^+^ calcd for C_15_H_9_Cl_3_N_4_O: 366.9915, Found: 366.9911.

#### 3.5.17. N-(2-Trichloromethylquinazolin-4-yl)acetamide (**20**)

Yield 31%. White solid. Mp 217 °C, (isopropanol). ^1^H NMR (250 MHz, CDCl_3_) δ = 8.54 (br s, 1H), 8.15–8.19 (m, 1H), 7.96–8.03 (m, 2H), 7.73–7.80 (m, 1H), 2.85 (s, 3H). ^13^C NMR (63 MHz, CDCl_3_) δ = 173.8, 159.9, 157.0, 150.4, 134.8, 130.0, 129.4, 122.0, 114.3, 97.0, 26.7. LC-MS (ESI+) *t_R_* 3.36 min, *m*/*z* [M + H]^+^ 303.98/305.91/307.99. MW: 304.56 g.mol^−1^. Anal. Calcd for C_11_H_8_Cl_3_N_3_O: C, 43.38; H, 2.65; N, 13.80. Found: C, 42.84; H, 2.59; N, 13.79.

#### 3.5.18. N-(2-Trichloromethylquinazolin-4-yl)propionamide (**21**)

Yield 52%. Yellow solid. Mp 171 °C, (isopropanol). ^1^H NMR (250 MHz, DMSO-*d*_6_) δ = 11.16 (s, 1H), 8.47 (d, *J* = 7.4 Hz, 1H), 8.27–7.95 (m, 2H), 7.91–7.68 (m, 1H), 3.03–2.74 (m, 2H), 1.30–1.00 (m, 3H). ^13^C NMR (63 MHz, DMSO-*d*_6_) δ = 175.2, 159.3, 158.8, 149.7, 135.4, 129.1, 128.6, 125.0, 115.4, 97.1, 30.4, 9.1. LC-MS (ESI+) *t_R_* 3.39 min, *m*/*z* [M + H]^+^ 317.98/320.00/322.08. MW: 318.59 g.mol^−1^. HRMS *m*/*z* [M + H]^+^ calcd for C_12_H_10_Cl_3_N_3_O: 317.9967, Found: 317.9964.

#### 3.5.19. N-(2-Trichloromethylquinazolin-4-yl)isobutyramide (**22**)

Yield 52%. White solid. Mp 161 °C, (isopropanol). ^1^H NMR (400 MHz, DMSO-*d*_6_) δ = 11.09 (s, 1H), 8.34 (d, *J* = 8.4 Hz, 1H), 8.09 (d, *J* = 3.7 Hz, 2H), 7.88–7.75 (m, 1H), 1.27–1.15 (m, 7H). ^13^C NMR (101 MHz, DMSO-*d*_6_) δ = 178.0, 159.4, 159.2, 149.8, 135.5, 129.1, 128.6, 125.3, 116.0, 97.1, 34.4, 19.3 (2C). LC-MS (ESI+) *t_R_* 3.56 min, *m*/*z* [M + H]^+^ 332.09/334.97/344.17. MW: 332.61 g.mol^−1^. HRMS *m*/*z* [M + H]^+^ calcd for C_13_H_12_Cl_3_N_3_O: 332.0119, Found: 332.0118.

#### 3.5.20. N-(2-Trichloromethylquinazolin-4-yl)pivalamide (**23**)

Yield 55%. White solid. Mp 157 °C, (isopropanol). ^1^H NMR (400 MHz, DMSO-*d*_6_) δ = 10.73 (s, 1H), 8.17–8.08 (m, 2H), 8.01–7.94 (m, 1H), 7.88–7.80 (m, 1H), 1.39 (s, 9H). ^13^C NMR (101 MHz, DMSO-*d*_6_) δ = 178.0, 161.0, 159.6, 150.1, 135.6, 129.3, 128.5, 126.3, 118.5, 96.9, 26.8, 26.3 (3C). LC-MS (ESI+) *t_R_* 3.24 min, *m*/*z* [M + H]^+^ 346.08/348.13/350.10. MW: 346.64 g.mol^−1^. HRMS *m*/*z* [M + H]^+^ calcd for C_14_H_14_Cl_3_N_3_O: 346.0275, Found: 346.0276.

#### 3.5.21. N-(2-Trichloromethylquinazolin-4-yl)pentanamide (**24**)

Yield 43%. Yellow solid. Mp 136 °C, (isopropanol). ^1^H NMR (400 MHz, DMSO-*d*_6_) δ = 11.16 (s, 1H), 8.47 (d, *J* = 8.4 Hz, 1H), 8.10–8.04 (m, 2H), 7.88–7.79 (m, 1H), 2.92 (t, *J* = 7.6 Hz, 2H), 1.65 (dt, *J* = 15.1, 7.5 Hz, 2H), 1.38 (dq, *J* = 14.6, 7.3 Hz, 2H), 0.91 (t, *J* = 7.4 Hz, 3H). ^13^C NMR (101 MHz, DMSO-*d*_6_) δ = 174.3, 159.3, 158.8, 149.7, 135.3, 129.0, 128.6, 125.0, 115.4, 97.2, 36.7, 26.6, 21.8, 13.7. LC-MS (ESI+) *t_R_* 4.26 min, *m*/*z* [M + H]^+^ 346.08/348.11/349.98. MW: 346.64 g.mol^−1^. HRMS *m*/*z* [M + H]^+^ calcd for C_14_H_14_Cl_3_N_3_O: 346.0275, Found: 346.0274.

#### 3.5.22. N-(2-Trichloromethylquinazolin-4-yl)cyclohexanecarboxamide (**25**)

Yield 51%. White solid. Mp 214 °C, (isopropanol). ^1^H NMR (250 MHz, DMSO-*d*_6_) δ = 11.05 (s, 1H), 8.37 (d, *J* = 8.4 Hz, 1H), 8.08 (d, *J* = 3.7 Hz, 2H), 7.88–7.78 (m, 1H), 2.06–1.92 (m, 2H), 1.84–1.62 (m, 3H), 1.53–1.18 (m, 6H). ^13^C NMR (63 MHz, DMSO-*d*_6_) δ = 177.2, 167.9, 159.2, 149.7, 135.4, 129.1, 128.6, 125.2, 115.7, 97.1, 43.8, 28.9 (2C), 25.4, 25.2 (2C). LC-MS (ESI+) *t_R_* 4.58 min, *m*/*z* [M + H]^+^ 372.07/374.11/376.06. MW: 372.68 g.mol^−1^. HRMS *m*/*z* [M + H]^+^ calcd for C_16_H_16_Cl_3_N_3_O: 372.0432, Found: 372.0428.

### 3.6. General Procedure for the Preparation of Compounds (**26**–**44**)

A mixture of 4-chloro-2-trichloromethylquinazoline (**4**) (0.2 g, 0.71 mmol), DMAP (26 mg, 0.21 mmol, 0.3 equiv.), and adequate alcohol derivative (0.85 mmol, 1.2 equiv.) in toluene (3 mL) was placed in a miniaturized sealed reactor (5 mL). The reaction mixture was irradiated in a monomode microwave oven, for 1 h at 130 °C. After removal of the toluene under reduced pressure, the residue was purified by silica gel column chromatography and recrystallized from appropriate solvent.

#### 3.6.1. 4-Methoxy-2-Trichloromethylquinazoline (**26**)

Yield 81%. White powder. Mp 83 °C, (isopropanol). ^1^H NMR (200 MHz, CDCl_3_) δ = 8.21 (d, *J* = 8 Hz, 1H), 8.08 (d, *J* = 8 Hz, 1H), 7.95–7.87 (m, 1H), 7.70–7.63 (m, 1H), 4.28 (s, 3H). ^13^C NMR (50 MHz, CDCl_3_) δ = 168.1, 160.2, 150.2, 146.6, 134.3, 128.7, 128.5, 123.5, 97.1, 54.9. LC-MS (ESI+) *t_R_* 4.46 min, *m*/*z* [M + H]^+^ 277.05/279.06/281.05. MW: 277.53 g/mol. Anal. Calcd for C_17_H_13_Cl_3_N_2_O: C, 43.28; H, 2.54; N, 10.09. Found: C, 44.17; H, 3.14; N, 9.54.

#### 3.6.2. 4-Ethoxy-2-Trichloromethylquinazoline (**27**)

Yield 79%. White powder. Mp 73 °C, (isopropanol). ^1^H NMR (200 MHz, CDCl_3_) δ = 8.23 (d, *J* = 8.2 Hz, 1H), 8.19 (d, *J* = 8.2 Hz, 1H), 7.95–7.85 (m, 1H), 7.68–7.61 (m, 1H), 4.75 (q, *J* = 8 Hz, 2H), 1.55 (t, *J* = 8 Hz, 3H). ^13^C NMR (50 MHz, CDCl_3_) δ = 167.8, 160.3, 150.3, 134.3, 128.7, 128.5, 123.7, 115.6, 97.2, 64.1, 14.2. LC-MS (ESI+) *t_R_* 4.92 min, *m*/*z* [M + H]^+^ 290.58/292.58/294.91. MW: 291.56 g/mol. Anal. Calcd for C_11_H_9_Cl_3_N_2_O: C, 45.31; H, 3.11; N, 9.61. Found: C, 45.16; H, 3.13; N, 9.65.

#### 3.6.3. 4-Propoxy-2-Trichloromethylquinazoline (**28**)

Yield 75%. Sand powder. Mp 39 °C, (methanol). ^1^H NMR (200 MHz, CDCl_3_) δ = 8.24 (d, *J* = 8.2 Hz, 1H), 8.07 (d, *J* = 8.2 Hz, 1H), 7.94–7.85 (m, 1H), 7.68–7.61 (m, 1H), 4.66 (t, *J* = 7.5 Hz, 2H), 1.97 (q, *J* = 7.5 Hz, 2H), 1.12 (t, *J* = 7.5 Hz, 3H). ^13^C NMR (50 MHz, CDCl_3_) δ = 167.6, 160.0, 150.2, 134.3, 128.7, 128.5, 123.6, 116.3, 97.2, 69.6, 22.0, 10.6. LC-MS (ESI+) *t_R_* 5.23 min, *m*/*z* [M + H]^+^ 305.05/307.03/309.06. MW: 305.59 g/mol. Anal. Calcd for C_12_H_11_Cl_3_N_2_O: C, 47.16; H, 3.63; N, 9.17. Found: C, 46.87; H, 3.59; N, 9.23.

#### 3.6.4. 4-Butoxy-2-Trichloromethylquinazoline (**29**)

Yield 79%. Yellow oil. ^1^H NMR (200 MHz, CDCl_3_) δ = 8.17 (d, *J* = 8.2 Hz, 1H), 8.03 (d, *J* = 8.2 Hz, 1H), 7.90–7.80 (m, 1H), 7.65–7.57 (m, 1H), 4.68 (t, *J* = 4.6 Hz, 2H), 1.97–1.83 (m, 2H), 1.64–1.46 (m, 2H), 1.01 (t, *J* = 7.4 Hz, 3H). ^13^C NMR (50 MHz, CDCl_3_) δ = 167.9, 160.3, 150.2, 134.2, 128.6, 128.5, 123.6, 115.5, 97.3, 67.9, 30.7,19.3, 13.9. LC-MS (ESI+) *t_R_* 5.42 min, *m*/*z* [M + H]^+^ 318.85/320.96/322.47. MW: 319.61 g/mol. Anal. Calcd for C_13_H_13_Cl_3_N_2_: C, 48.85; H, 4.10; N, 8.76. Found: C, 49.12; H, 4.04; N, 8.65.

#### 3.6.5. 4-Isopropoxy-2-Trichloromethylquinazoline (**30**)

Yield 53%. Grey powder. Mp 210 °C, (methanol). ^1^H NMR (200 MHz, CDCl_3_) δ = 8.20 (d, *J* = 8.2 Hz, 1H), 8.05 (d, *J* = 8.2 Hz, 1H), 7.93–7.85 (m, 1H), 7.68–7.60 (m, 1H), 5.69 (t, *J* = 6 Hz, 1H), 1.52 (d, *J* = 6 Hz, 6H). ^13^C NMR (50 MHz, CDCl_3_) δ = 167.4, 160.4, 150.3, 134.2, 128.7, 128.4, 123.7, 115.8, 97.3, 71.8, 21.7. LC-MS (ESI+) *t_R_* 5.24 min, *m*/*z* [M + H]^+^ 305.04/307.05/309.10. MW: 305.59 g/mol. Anal. Calcd for C_12_H_11_Cl_3_N_2_O: C, 47.16; H, 3.63; N, 9.17. Found: C, 46.81; H, 3.72; N, 9.39.

#### 3.6.6. 4-(Prop-2-ynyloxy)-2-Trichloromethylquinazoline (**31**)

Yield 87%. Beige powder. Mp 62 °C, (methanol). ^1^H NMR (200 MHz, CDCl_3_) δ = 8.26 (d, *J* = 8.2 Hz, 1H), 8.10 (d, *J* = 8.2 Hz, 1H), 7.97–7.89 (m, 1H), 7.73–7.65 (m, 1H), 5.31 (d, *J* = 2.5 Hz, 2H), 2.58 (t, *J* = 2.5 Hz, 1H). ^13^C NMR (50 MHz, CDCl_3_) δ = 166.7, 159.8, 150.4, 134.7, 128.9, 128.8, 123.6, 115.2, 96.9, 77.4, 75.7, 55.3. LC-MS (ESI+) *t_R_* 4.42 min, *m*/*z* [M + H]^+^ 301.02/303.03/305.04. MW: 301.56 g/mol. Anal. Calcd for C_12_H_7_Cl_3_N_2_O: C, 47.79; H, 2.34; N, 9.29. Found: C, 48.38; H, 2.25; N, 9.08.

#### 3.6.7. 4-(But-3-yn-2-yloxy)-2-Trichloromethylquinazoline (**32**)

Yield 89%. Beige powder. Mp 92 °C, (methanol). ^1^H NMR (200 MHz, CDCl_3_) δ = 8.26 (d, *J* = 8.2 Hz, 1H), 8.09 (d, *J* = 8.2 Hz, 1H), 7.96–7.91 (m, 1H), 7.72–7.64 (m, 1H), 6.18 (qd, *J* = 8 Hz, *J* = 2 Hz, 1H), 2.52 (d, *J* = 2 Hz, 1H), 1.80 (d, *J* = 8 Hz, 3H). ^13^C NMR (50 MHz, CDCl_3_) δ = 166.4, 159.9, 150.5, 134.6, 128.8, 128.7, 123.7, 115.4, 97.0, 81.9, 73.7, 63.7, 21.2. LC-MS (ESI+) *t_R_* 4.73 min, *m*/*z* [M + H]^+^ 315.02/317.04/318.98. MW: 315.58 g/mol. Anal. Calcd for C_13_H_9_Cl_3_N_2_O: C, 49.48; H, 2.87; N, 8.88. Found: C, 48.96; H, 2.95; N, 8.93.

#### 3.6.8. 4-(4-Methylpent-1-yn-3-yloxy)-2-Trichloromethylquinazoline (**33**)

Yield 90%. Beige powder. Mp 96 °C, (methanol). ^1^H NMR (200 MHz, CDCl_3_) δ = 8.30–8.27 (m, 1H), 8.09 (d, *J* = 8.2 Hz, 1H), 7.97–7.88 (m, 1H), 7.72–7.64 (m, 1H), 5.97 (dd, *J* = 6 Hz, *J* = 2 Hz, 1H), 2.49 (d, *J* = 2 Hz, 1H), 2.44–2.28 (m, 1H), 1.20 (d, *J* = 4 Hz, 3H), 1.17 (d, *J* = 4 Hz, 3H). ^13^C NMR (50 MHz, CDCl_3_) δ = 166.7, 160.0, 150.6, 134.6, 128.8, 128.7, 123.6, 115.5, 97.0, 79.5, 74.9, 72.1, 32.4, 18.2, 17.6. LC-MS (ESI+) *t_R_* 5.21 min, *m*/*z* [M + H]^+^ 343.17/345.22/347.09. MW: 343.64 g/mol. Anal. Calcd for C_15_H_13_Cl_3_N_2_O: C, 52.43; H, 3.81; N, 8.15. Found: C, 53.02; H, 3.75; N, 8.37.

#### 3.6.9. 2-(2-Trichloromethylquinazolin-4-yloxy)ethanol (**34**)

Yield 42%. White powder. Mp 93 °C, (methanol). ^1^H NMR (200 MHz, CDCl_3_) δ = 8.31–8.28 (m, 1H), 8.15 (d, *J* = 8.2 Hz, 1H), 8.01–7.93 (m, 1H), 7.78–7.70 (m, 1H), 4.94–4.91 (m, 2H), 4.21–4.17 (m, 2H), 2.49 (br s, 1H). ^13^C NMR (50 MHz, CDCl_3_) δ = 168.1, 159.8, 150.3, 134.8, 129.0, 128.8, 123.7, 115.4, 97.1, 70.1, 61.8. LC-MS (ESI+) *t_R_* 3.33 min, *m*/*z* [M + H]^+^ 306.93/308.91/311.07. MW: 307.56 g/mol. Anal. Calcd for C_11_H_9_Cl_3_N_2_O: C, 42.96; H, 2.95; N, 9.11. Found: C, 43.71; H, 3.03; N, 8.95.

#### 3.6.10. 4-(2-Methoxyethoxy)-2-Trichloromethylquinazoline (**35**)

Yield 97%. White powder. Mp 63 °C, (methanol). ^1^H NMR (200 MHz, CDCl_3_) δ = 8.31 (d, *J* = 8 Hz, 1H), 8.15 (d, *J* = 8 Hz, 1H), 7.91 (t, *J* = 6 Hz, 1H), 7.66 (t, *J* = 6 Hz, 1H), 4.87–4.83 (m, 2H), 3.92–3.88 (m, 2H), 3.47 (s, 3H). ^13^C NMR (50 MHz, CDCl_3_) δ = 167.7, 160.2, 150.6, 134.2, 128.8, 128.4, 123.7, 115.5, 97.2, 70.4, 66.9, 59.0. LC-MS (ESI+) *t_R_* 4.28 min, *m*/*z* [M + H]^+^ 321.00/322.91/325.13. MW: 321.59 g/mol. Anal. Calcd for C_12_H_11_Cl_3_N_2_O: C, 44.82; H, 3.45; N, 8.71. Found: C, 44.35; H, 3.38; N, 8.92.

#### 3.6.11. 4-(2-Chloroethoxy)-2-Trichloromethylquinazoline (**36**)

Yield 79%. Sand powder. Mp 54 °C, (methanol). ^1^H NMR (200 MHz, CDCl_3_) δ = 8.26 (d, *J* = 8 Hz, 1H), 8.09 (d, *J* = 8 Hz, 1H), 7.97–7.89 (m, 1H), 7.73–7.65 (m, 1H), 4.94 (t, *J* = 6 Hz, 2H), 4.00 (t, *J* = 6 Hz, 2H). ^13^C NMR (50 MHz, CDCl_3_) δ = 167.3, 159.9, 150.4, 134.7, 128.9, 128.8, 123.6, 115.2, 96.9, 67.4, 41.4. LC-MS (ESI+) *t_R_* 4.60 min, *m*/*z* [M + H]^+^ 325.03/327.01/329.07. MW: 326.01 g/mol. Anal. Calcd for C_11_H_8_Cl_4_N_2_O: C, 40.53; H, 2.47; N, 8.59. Found: C, 43.89; H, 3.45; N, 9.02.

#### 3.6.12. 4-(3-Chloropropoxy)-2-Trichloromethylquinazoline (**37**)

Yield 70%. Beige powder. Mp 77 °C, (methanol). ^1^H NMR (200 MHz, CDCl_3_) δ = 8.22 (d, *J* = 8 Hz, 1H), 8.08 (d, *J* = 8 Hz, 1H), 7.96–7.88 (m, 1H), 7.71–7.64 (t, *J* = 6 Hz, 1H), 4.86 (t, *J* = 6 Hz, 2H), 3.79 (t, *J* = 6 Hz, 2H), 2.42 (t, *J* = 6 Hz, 2H). ^13^C NMR (50 MHz, CDCl_3_) δ = 167.6, 160.1, 150.3, 134.5, 128.9, 128.7, 123.5, 115.4, 97.1, 64.7, 41.3, 31.6. LC-MS (ESI+) *t_R_* 4.89 min, *m*/*z* [M + H]^+^ 339.04/341.06/343.09. MW: 340.03 g/mol. Anal. Calcd for C_12_H_10_Cl_4_N_2_O: C, 42.39; H, 2.96; N, 8.24. Found: C, 42.06; H, 3.01; N, 8.51.

#### 3.6.13. 4-(4-Chlorobutoxy)-2-Trichloromethylquinazoline (**38**)

Yield 28%. Yellow oil. ^1^H NMR (200 MHz, CDCl_3_) δ = 8.20 (d, *J* = 8 Hz, 1H), 8.07 (d, *J* = 8 Hz, 1H), 7.95–7.87 (m, 1H), 7.66 (t, *J* = 6 Hz, 1H), 4.74 (t, *J* = 6 Hz, 2H), 3.66 (t, *J* = 6 Hz, 2H), 2.00–2.15 (m, 4H). ^13^C NMR (50 MHz, CDCl_3_) δ = 167.7, 160.2, 150.3, 134.4, 128.8, 128.7, 123.5, 115.4, 97.2, 67.2, 44.6, 29.3, 26.1. LC-MS (ESI+) *t_R_* 5.09 min, *m*/*z* [M + H]^+^ 352.94/354.55/357.10. MW: 354.06g/mol. Anal. Calcd for C_13_H_12_Cl_4_N_2_O: C, 44.10; H, 3.42; N, 7.91. Found: C, 43.87; H, 3.39; N, 8.02.

#### 3.6.14. 4-(2-Fluoroethoxy)-2-Trichloromethylquinazoline (**39**)

Yield 71%. Off-white powder. Mp 73 °C, (isopropanol). ^1^H NMR (200 MHz, CDCl_3_) δ = 8.22 (d, *J* = 8 Hz, 1H), 8.05 (d, *J* = 8 Hz, 1H), 7.92–7.86 (m, 1H), 7.64 (t, *J* = 6 Hz, 1H), 5.00 (s, 2H), 4.81–4.89 (m, 2H). ^13^C NMR (50 MHz, CDCl_3_) δ = 167.3, 159.8, 150.3, 134.7, 128.8, 128.7, 123.6, 115.2, 97.1, 81.2 (d, *J* C-F = 171 Hz), 66.8 (d, *J* C-F = 21 Hz). LC-MS (ESI+) *t_R_* 4.17 min, *m*/*z* [M + H]^+^ 309.15/311.13/313.16. MW: 309.55 g/mol. Anal. Calcd for C_11_H_8_Cl_3_FN_2_O: C, 42.68; H, 2.60; N, 9.05. Found: C, 42.87; H, 2.55; N, 8.93.

#### 3.6.15. 4-(2-Bromoethoxy)-2-Trichloromethylquinazoline (**40**)

Yield 71%. Beige powder. Mp 100 °C, (isopropanol). ^1^H NMR (200 MHz, CDCl_3_) δ = 8.30 (d, *J* = 8 Hz, 1H), 7.84–7.83 (m, 2H), 7.66–7.61 (m, 1H), 4.85–4.78 (m, 2H), 3.92–3.85 (m, 2H). ^13^C NMR (50 MHz, CDCl_3_) δ = 162.6, 147.6, 144.1, 135.2, 129.4, 128.8, 127.1, 102.6, 93.4, 47.7, 38.8. LC-MS (ESI+) *t_R_* 4.73 min, *m*/*z* [M + H]^+^ 369.12/370.96/373.11. MW: 370.46 g/mol. Anal. Calcd for C_11_H_8_BrCl_3_N_2_O: C, 35.66; H, 2.18; N, 7.56. Found: C, 35.92; H, 2.29; N, 7.37.

#### 3.6.16. N,N-Diethyl-2-[(2-Trichloromethylquinazolin-4-yl)oxy]ethanamine (**41**)

Yield 67%. Off-white powder. Mp 137 °C, (isopropanol). ^1^H NMR (200 MHz, CDCl_3_) δ = 8.24 (d, *J* = 8 Hz, 1H), 8.21–8.20 (m, 1H), 8.10–8.06 (m, 1H), 7.71–7.67 (m, 1H), 4.77 (t, *J* = 6 Hz, 4H), 3.02 (t, *J* = 6 Hz, 4H), 2.69 (q, *J* = 7 Hz, 4H), 1.09 (t, *J* = 7 Hz, 6H). ^13^C NMR (50 MHz, CDCl_3_) δ = 167.8, 160.3, 150.6, 134.2, 128.8, 128.4, 123.6, 115.5, 97.3, 66.2, 51.1, 48.0, 11.9. LC-MS (ESI+) *t_R_* 4.53 min, *m*/*z* [M + H]^+^ 362.17/364.21/366.18. MW: 362.68 g/mol. Anal. Calcd for C_15_H_18_Cl_3_N_3_O: C, 49.67; H, 5.00; N, 11.59. Found: C, 49.19; H, 5.11; N, 11.75.

#### 3.6.17. N-{2-[(2-Trichloromethylquinazolin-4-yl)oxy]ethyl}acetamide (**42**)

Yield 79%. White powder. Mp 135 °C, (isopropanol). ^1^H NMR (200 MHz, CDCl_3_) δ = 8.27 (d, *J* = 8 Hz, 1H), 8.13 (d, *J* = 8 Hz, 1H), 7.99 (t, *J* = 7 Hz, 1H), 7.74 (t, *J* = 7 Hz, 1H), 6.61 (br s, 1H), 4.87 (t, *J* = 5 Hz, 2H), 3.88 (d, *J* = 5 Hz, 2H), 2.07 (s, 3H). ^13^C NMR (50 MHz, CDCl_3_) δ = 171.9, 167.2, 159.8, 150.3, 134.8, 129.0, 128.8, 123.7, 115.2, 97.0, 66.4, 39.6, 22.6. LC-MS (ESI+) *t_R_* 3.38 min, *m*/*z* [M + H]^+^ 348.22/350.14/351.93. MW: 348.61 g/mol. Anal. Calcd for C_13_H_12_Cl_3_N_3_O_2_: C, 44.79; H, 3.47; N, 12.05. Found: C, 45.23; H, 3.39; N, 12.35.

#### 3.6.18. 4-[2-(Piperidin-1-yl)ethoxy]-2-Trichloromethylquinazoline (**43**)

Yield 20%. Yellow oil. ^1^H NMR (200 MHz, CDCl_3_) δ = 8.13–8.26 (m, 1H), 7.99–8.11 (m, 1H), 7.76–7.99 (m, 1H), 7.57–7.74 (m, 1H), 4.82 (t, *J* = 6.1 Hz, 2H), 2.91 (t, *J* = 6.1 Hz, 2H), 2.49–2.62 (m, 4H), 1.50–1.69 (m, 4H), 1.42 (dt, *J* = 19.8, 7.3 Hz, 2H). ^13^C NMR (50 MHz, CDCl_3_) δ = 167.8, 160.3, 150.4, 134.4, 128.8, 128.7, 123.8, 115.6, 97.3, 65.9, 57.3, 55.1, 26.1, 24.3. LC-MS (ESI+) *t_R_* 4.65 min, *m*/*z* [M + H]^+^ 374.05/376.08/378.05. MW: 374.69 g/mol. HRMS *m*/*z* [M + H]^+^ calcd for C_16_H_18_Cl_3_N_3_O: 374.0588, found: 374.0588.

#### 3.6.19. 4-[2-(Pyrrolidin-1-yl)ethoxy]-2-Trichloromethylquinazoline (**44**)

Yield 47%. Yellow oil. ^1^H NMR (200 MHz, CDCl_3_) δ = 8.25 (d, *J* = 8.3 Hz, 1H), 8.07 (d, *J* = 8.3 Hz, 1H), 7.84–7.97 (m, 1H), 7.58–7.78 (m, 1H), 5.02 (t, *J* = 5.5 Hz, 2H), 3.33 (t, *J* = 5.5 Hz, 2H), 2.91–3.18 (m, 4H), 1.82–2.09 (m, 4H). ^13^C NMR (50 MHz, CDCl_3_) δ = 167.3, 159.9, 150.4, 134.8, 129.1, 128.9, 123.7, 115.3, 97.1, 64.9, 54.7, 53.7, 23.5. LC-MS (ESI+) *t_R_* 3.77 min, *m*/*z* [M + H]^+^ 360.03/362.15/364.10. MW: 360.67 g/mol. HRMS *m*/*z* [M + H]^+^ calcd for C_15_H_16_Cl_3_N_3_O: 360.0432, found: 360.0429.

### 3.7. General Procedure for the Preparation of Compounds (**45**–**46**)

To a solution of 4-methoxybenzamide (1.5 equiv.) in dry DMF (3 mL) at 0 °C under N_2_, 60% sodium hydride in oil (1.5 equiv.) were added portion wise. The resulting mixture were added dropwise to a solution of 4-chloro-2-methylquinazoline or 4-chloro-2-trifluoromethylquinazoline (200 mg, 1.0 equiv.) in dry DMF (2 mL) at 0 °C under N_2_. The reaction was stirred overnight at rt. Then, the excess of NaH was hydrolyzed with ice. The reaction mixture was extracted with EtOAc and washed three times with brine. The organic layer was dried with Na_2_SO_4_, filtered and concentrated. The crude product was purified by silica gel column chromatography and recrystallized from isopropanol to give the corresponding compound.

#### 3.7.1. 4-methoxy-N-(2-Methylquinazolin-4-yl)benzamide (**45**)

Yield 15%. Yellow solid. Mp 169 °C, (isopropanol). ^1^H NMR (400 MHz, CDCl_3_) δ =14.96 (s, 1H), 8.71 (d, *J* = 7.7 Hz, 1H), 8.39 (d, *J* = 8.7 Hz, 2H), 7.87–7.78 (m, 1H), 7.72 (d, *J* = 8.1 Hz, 1H), 7.60–7.52 (m, 1H), 6.98 (d, *J* = 8.9 Hz, 2H), 3.89 (s, 3H), 2.63 (s, 3H). ^13^C NMR (101 MHz, CDCl_3_) δ 179.7, 163.2, 158.1, 150.9, 148.8, 135.1, 131.9, 130.0, 127.1, 126.1, 119.8, 113.5 (4C), 55.4, 22.8. LC-MS (ESI+) *t_R_* 2.58 min, *m*/*z* [M + H]^+^ 294.10. MW: 293.32 g.mol^−1^. HRMS *m*/*z* [M + H]^+^ calcd for C_17_H_15_N_3_O_2_: 294.1237, Found: 294.1234.

#### 3.7.2. 4-methoxy-N-(2-Trifluoromethylquinazolin-4-yl)benzamide (**46**)

Yield 17%. White solid. Mp 190 °C, (isopropanol). ^1^H NMR (400 MHz, DMSO-*d*_6_) δ = 11.53 (s, 1H), 8.26 (d, *J* = 8.4 Hz, 1H), 8.20–8.10 (m, 2H), 8.07 (d, *J* = 8.9 Hz, 2H), 7.90–7.81 (m, 1H), 7.11 (d, *J* = 8.9 Hz, 2H), 3.87 (s, 3H). ^13^C NMR (101 MHz, DMSO-*d*_6_) δ 166.68, 162.99, 161.01, 150.71 (q, *J* = 35.6 Hz), 150.38, 135.65, 130.98 (2C), 129.41, 128.46, 126.39, 125.06, 119.79 (q, *J* = 275.6 Hz), 118.76, 113.80 (2C), 55.57. LC-MS (ESI+) *t_R_* 2.53 min, *m*/*z* [M + H]^+^ 348.29. MW: 347.29 g.mol^−1^. HRMS *m*/*z* [M + H]^+^ calcd for C_17_H_12_F_3_N_3_O_2_: 348.0954, Found: 348.0953.

### 3.8. General Procedure for the Preparation of Compounds (**47**–**49**)

A mixture of 4-chloro-2-methylquinazoline, 4-chloro-2-trifluoromethylquinazoline or 4-chloroquinazoline (400 mg, 1 equiv.), DMAP (1.1 equiv) and 2-(diethylamino)ethanol (2 equiv.) in toluene (3 mL) was placed in a miniaturized sealed reactor (5 mL). The reaction mixture was irradiated in a monomode microwave oven, for 1 h at 130 °C. After removal of the toluene under reduced pressure, the residue was purified by silica gel column chromatography, deactivated by triethylamine, and recrystallized from isopropanol.

#### 3.8.1. N,N-Diethyl-2-[(2-Methylquinazolin-4-yl)oxy]ethanamine (**47**)

Yield 39%. Yellow powder. Mp 230 °C, (isopropanol). ^1^H NMR (200 MHz, CDCl_3_) δ = 7.98–7.95 (m, 1H), 7.73–7.62 (m, 2H), 7.35–7.29 (1H, m), 4.55–4.51 (2H, m), 2.89–2.84 (2H, m), 2.61–2.53 (7H, m), 1.02–0.97 (6H, m). 13C NMR (50 MHz, CDCl_3_) δ = 166.2, 163.6, 151.2, 133.1, 126.7, 125.8, 123.2, 114.4, 64.9, 50.8, 47.8(2C), 26.2, 11.9(2C). LC-MS (ESI+) *t_R_* 1.82 min, *m*/*z* [M + H]^+^ 260.55. MW: 259.35 g/mol. Anal. Calcd for C_15_H_21_N_3_O: C, 69.47; H, 8.16; N, 16.20. Found: C, 70.05; H, 8.07; N, 16.39.

#### 3.8.2. N,N-Diethyl-2-[(2-Trifluoromethylquinazolin-4-yl)oxy]ethan-1-Amine (**48**)

Yield 40%. Colorless oil. ^1^H NMR (400 MHz, CDCl_3_) δ = 8.23 (dd, *J* = 8.2, 0.9 Hz, 1H), 8.07 (d, *J* = 8.4 Hz, 1H), 7.97–7.88 (m, 1H), 7.72–7.65 (m, 1H), 4.75 (t, J = 6.2 Hz, 2H), 3.00 (t, *J* = 6.2 Hz, 2H), 2.74–2.63 (m, 4H), 1.16–1.01 (m, 6H). ^13^C NMR (101 MHz, CDCl_3_) δ 168.2, 152.1 (q, *J* = 36.4 Hz), 150.5, 134.6, 128.9, 128.7, 123.9, 119.8 (q, *J* = 275.6 Hz), 116.8, 66.6, 50.9, 48.1 (2C), 12.2 (2C). LC-MS (ESI+) *t_R_* 2.84 min, *m*/*z* [M + H]^+^ 314.18. MW: 313.32 g/mol. HRMS *m*/*z* [M + H]^+^ calcd for C_15_H_18_F_3_N_3_O: 314.1475, Found: 314.1475.

#### 3.8.3. N,N-Diethyl-2-(Quinazolin-4-yloxy)ethanamine (**49**)

Yield 10%. Yellow oil. ^1^H NMR (400 MHz, CDCl_3_) δ = 8.77 (s, *J* = 5.6 Hz, 1H), 8.18–8.05 (m, 1H), 7.90 (d, *J* = 8.4 Hz, 1H), 7.85–7.75 (m, 1H), 7.59–7.44 (m, 1H), 4.64 (t, *J* = 6.1 Hz, 2H), 2.98 (t, *J* = 6.1 Hz, 2H), 2.66 (q, *J* = 7.1 Hz, 4H), 1.08 (t, *J* = 7.1 Hz, 6H). ^13^C NMR (101 MHz, CDCl_3_) δ = 166.8, 154.5, 151.0, 133.6, 127.7, 127.1, 123.6, 116.8, 65.6, 51.1, 47.9, 12.1. LC-MS (ESI+) *t_R_* 1.11 min, *m*/*z* [M + H]^+^ 246.21. MW: 245.32 g/mol. Anal. Calcd for C_14_H_19_N_3_O: C, 43.28; H, 2.54; N, 10.09. Found: C, 44.17; H, 3.14; N, 9.54.

### 3.9. Biology

#### 3.9.1. In Vitro Cytotoxicity Evaluation

HepG2 cell line was maintained at 37 °C, 5% CO_2_, at 90% humidity in MEM supplemented with 10% fetal bovine serum, 1% L-glutamine (200 mM) and penicillin (100 U/mL)/streptomycin (100 µg/mL) (complete RPMI medium). The cytotoxicity of the tested molecules on the HepG2 (hepatocarcinoma cell line purchased from ATCC, ref HB-8065) cell line was assessed according to the method of Mosmann [19] with slight modifications. Briefly, 5.10^3^ cells in 100 µL of complete medium were inoculated into each well of 96-well plates and incubated at 37 °C in humidified 5% CO_2_. After 24 h incubation, 100 μL of medium with various product concentrations dissolved in DMSO (final concentration less than 0.5% *v*/*v*) were added and the plates were incubated for 72 h at 37 °C. Triplicate assays were performed for each sample. Each plate-well was then microscope-examined for possible precipitate formation before the medium was aspirated from the wells. 100 μL of MTT (3-(4,5-dimethyl-2-thiazolyl)-2,5-diphenyl-2*H*-tetrazolium bromide) solution (0.5 mg/mL in medium without FBS) were then added to each well. Cells were incubated for 2 h at 37 °C. After this time, the MTT solution was removed and DMSO (100 μL) was added to dissolve the resulting blue formazan crystals. Plates were shaken vigorously (700 rpm) for 10 min. The absorbance was measured at 570 nm with 630 nm as reference wavelength using a BIO-TEK ELx808 Absorbance Microplate Reader (LabX, Midland, ON, Canada). DMSO was used as blank and doxorubicin (purchased from Sigma Aldrich) as positive control. Cell viability was calculated as percentage of control (cells incubated without compound). The 50% cytotoxic concentration (CC_50_) was determined from the dose–response curve, using TableCurve software 2D v.5.0. CC_50_ values represent the mean value calculated from three independent experiments.

#### 3.9.2. In Vitro Antiplasmodial Evaluation

In this study, a K1 culture-adapted *P. falciparum* strain resistant to chloroquine, pyrimethamine, and proguanil was used in an in vitro culture. It was maintained in continuous culture as described previously by Trager and Jensen [20]. Cultures were maintained in fresh A+ human erythrocytes at 2.5% hematocrit in complete medium (RPMI 1640 with 25 mM HEPES, 25 mM NaHCO_3_, 10% of A+ human serum) at 37 °C under reduced O_2_ atmosphere (gas mixture 10% O_2_, 5% CO_2_, and 85% N_2_). Parasitemia was maintained daily at between 1 and 3%. The *P. falciparum* drug susceptibility test was carried out by comparing quantities of DNA in treated and control cultures of parasite in human erythrocytes according to an SYBR Green I fluorescence-based method [21] using a 96-well fluorescence plate reader. Compounds, previously dissolved in DMSO (final concentration less than 0.5% *v*/*v*), were incubated in a total assay volume of 200 µL (RPMI, 2% hematocrit and 0.4% parasitemia) for 72 h in a humidified atmosphere (10% O_2_ and 5% CO_2_) at 37 °C, in 96-well flat bottom plates. Duplicate assays were performed for each sample. After incubation, plates were frozen at -20 °C for 24 h. Then, the frozen plates were thawed for 1 h at 37 °C. Fifteen μL of each sample were transferred to 96-well flat bottom non-sterile black plates (Greiner Bio-one) already containing 15 μL of the SYBR Green I lysis buffer (2X SYBR Green I, 20 mM Tris base pH 7.5, 20 mM EDTA, 0.008% w/v saponin, 0.08% w/v Triton X-100). Negative control treated by solvents (DMSO or H_2_O) and positive controls (chloroquine and doxycycline) were added to each set of experiments. Plates were incubated for 15 min at 37 °C and then read on a TECAN Infinite F-200 spectrophotometer with excitation and emission wavelengths at 485 and 535 nm, respectively. The concentrations of compounds required to induce a 50% decrease of parasite growth (EC_50_ K1) were calculated from three independent experiments.

## 4. Conclusions

From previously identified antiplasmodial hit **C** and series **D** in 4-substituted-2-trichloromethylquinazoline series, new derivatives were synthesized to explore the introduction of various 4-carboxamido or 4-alkoxy substituents. Thus, after screening reaction conditions to obtain carboxamide derivatives, 23 new molecules were obtained, including a dibenzamide. The four new hit molecules afforded are evidence that replacing a sulfonamido linker by a carboxamido one fosters antiplasmodial activity. In the second series, we obtained 19 new molecules bearing various alkoxy chains at position 4. Two new hits were obtained, both bearing a tertiary amine function in the alkoxy chain, like chloroquine. For the more potent molecules in each series, the analogs prepared without the 2-trichloromethyl group showed that this group is essential to activity against *P. falciparum*. This work supports search for new derivatives centered on the 2-trichloromethylquinazoline scaffold.

## Figures and Tables

**Table 1:** Structures and in vitro activities of previously described compounds **A**–**C** and series **D**.

	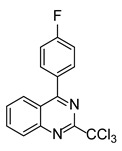	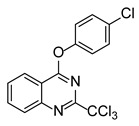	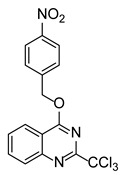	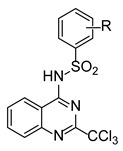
	A	B	C	Series D
Antiplasmodial activity EC_50_ *P. falciparum K1* (µM)	2.5	1.1	1.8	>10
Cytotoxicity CC_50_ *HepG2* (µM)	>125	50	19.4	38–136
Selectivity Index (SI)	>50	45	11	-

Reference molecules: Chloroquine (EC_50_ = 0.8 µM, CC_50_ = 30, SI = 37.5), Doxycycline (EC_50_ = 6 µM, CC_50_ = 20, SI = 3.3), Doxorubicin (CC_50_ = 0.2 µM). Selectivity index = CC_50_/EC_50._

**Table 2:** Studied parameters for the reaction of (**1**) with 4-chlorobenzoyl chloride.

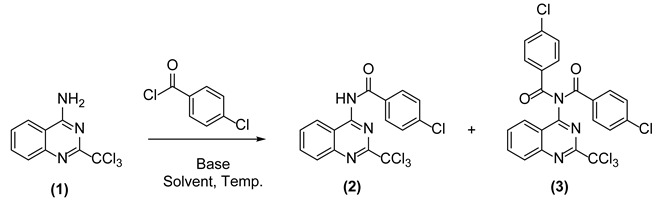						
N°	Base (equiv.)	Acyl Chloride (equiv.)	Solvent	Temp.	Time	LC-MS Estimated Conversion
1	NaH (1.0 equiv)	1.0 equiv	DMF	0 °C → R. T	12 h	(**1**) 80%, (**3**) 20%
2	NaH (1.0 equiv)	1.0 equiv	DMF	0 °C	24 h	(**1**) 95%, (**3**) 5%
3	NaH (1.5 equiv)	1.5 equiv	DMF	0 °C → R. T	24 h	(**1**) 75%, (**3**) 25%
4	NaH (2.0 Equiv)	2.0 equiv	DMF	0 °C → R. T	12 h	(**1**) 60%, (**3**) 40%
5	NaH (1.0 Equiv)	1.0 equiv	THF	0 °C → R. T	12 h	(**1**) 90%, (**3**) 10%
6	*t*BuOK (1.1 equiv)	1.5 equiv	DMF	0 °C → R. T	16 h	(**1**) 85%, (**3**) 10%
7	NaHMDS (1.1 equiv)	1.0 equiv	THF	0 °C → R. T	16 h	(**1**) 50%, (**2**) <5%, (**3**) 45%
8	Et_3_N (2.0 equiv)	3.0 equiv	Dioxane	105 °C	30 min	(**1**) >95%
9	Et_3_N (5.0 equiv)	3.0 equiv	Dioxane	105 °C	24 h	(**1**) >90%
10	NaH (5.0 Equiv)	2.0 equiv	DMF	0 °C → R. T	24 h	(**3**) 90% *

* Isolated yield.

**Table 3:** Structures, reaction yields, in vitro antiplasmodial and cytotoxicity evaluations of the 4-carboxamide-2-trichloro-methylquinazolines series (**2**–**3**, **5**–**25**).

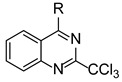					
Molecule	R-	Yield (%)	HepG2 CC_50_ (µM)	P*f*K1 EC_50_ (µM)	SI ^d^
**3**	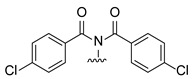	90	20.0	1.46	13.7
**5**	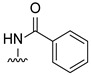	60	24.2	1.76	13.8
**6**	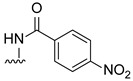	67	22.1	4.18	5.3
**7**	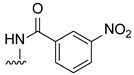	60	24.8	3.37	7.4
**8**	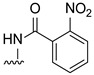	54	>15.6 ^c^	3.0	>5.2
**9**	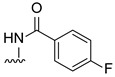	**88**	**29.4**	**1.34**	**21.9**
**10**	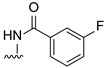	80	19.9	2.86	6.9
**11**	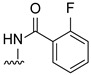	98	>15.6 ^c^	5.23	>3.0
**2**	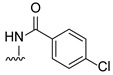	**74**	**21.0**	**0.99**	**21.2**
**12**	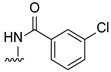	82	16.4	1.54	10.6
**13**	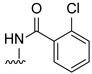	83	27.3	9.04	4.1
**14**	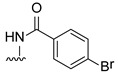	62	19.5	1.30	15.0
**15**	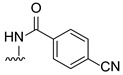	95	31.4	3.50	9.0
**16**	** 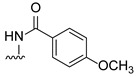 **	**75**	**27.7**	**0.94**	**29.5**
**17**	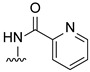	81	>7.8 ^c^	14.5	>0.5
**18**	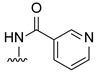	31	72.9	3.9	18.7
**19**	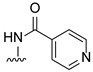	22	22.8	1.8	12.7
**20**	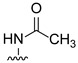	31	31.2	>10	>3.1
**21**	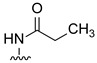	32	>62.5 ^c^	4.9	>12.8
**22**	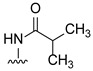	52	>31.2 ^c^	3.5	>9.0
**23**	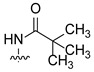	55	34.9	1.8	19.4
**24**	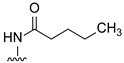	43	**42.3**	**1.6**	**26.4**
**25**	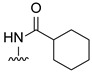	51	>15.6 ^c^	2.4	>6.5
**Doxorubicin ^a^**			0.2	-	-
**Chloroquine ^b^**			30	0.8	37.5
**Doxycycline ^b^**			20	6.0	3.3

^a^ Doxorubicin was used as a cytotoxic reference-drug; ^b^ Chloroquine and doxycycline were used as antimalarial reference-drugs; ^c^ CC_50_ could not be determined because of a lack of solubility of the tested molecule in the culture medium; ^d^ Selectivity index (SI) was calculated according to the formula: SI = CC_50_/EC_50_; In bold: hit-compounds (EC_50_ ≤ 2 µM and SI ≥ 20).

**Table 4:** Structures, reaction yields, in vitro antiplasmodial and cytotoxicity evaluations of the 4-alkoxy-2-trichloromethylquinazolines (**26**–**44).**

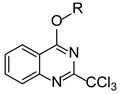					
Molecule	R-	Yield (%)	HepG2 CC_50_ (µM)	P*f*K1 EC_50_ (µM)	SI ^e^
**26**	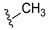	81	39.3	7.1	5.5
**27**	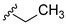	79	47.1	4.7	10
**28**	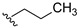	75	54.8	3.5	15
**29**	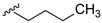	79	82.4	5.8	14.2
**30**	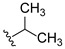	53	101.5	>10 ^d^	<10
**31**	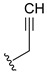	87	49.6	3.4	14.6
**32**	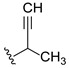	89	45.0	>10 ^d^	<4.5
**33**	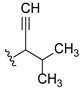	90	30.6	>10 ^d^	<3
**34**	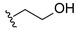	42	7.9	10.0	0.8
**35**	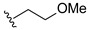	97	53.2	2.3	23.1
**36**	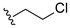	79	>62.5	2.2	>28
**37**	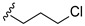	70	60.4	4.2	14.4
**38**	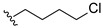	28	50.5	4.3	11.7
**39**	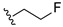	71	29.5	8.0	3.7
**40**	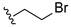	71	>62.5 ^c^	2.2	>28
**41**	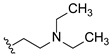	**67**	**32.3**	**0.9**	**35.9**
**42**	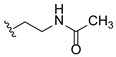	79	>99.7 ^c^	3.3	>30
**43**	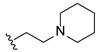	20	23.4	1.4	16.5
**44**	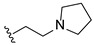	47	**43.7**	**1.3**	**33.6**
					
**Doxorubicin ^a^**			0.2	-	-
**Chloroquine ^b^**			30	0.8	37.5
**Doxycycline ^b^**			20	6	3.3

^a^ Doxorubicin was used as a cytotoxic reference-drug; ^b^ Chloroquinine and doxycycline were used as antimalarial reference-drugs; ^c^ CC_50_ could not be determined because of a lack of solubility of the tested molecule in the culture medium; ^d^ EC_50_ was not reached at the highest tested concentration (10 or 50 µM). ^e^ Selectivity index (SI) was calculated according to the formula: SI = CC_50_/EC_50_; In bold: hit-compounds (EC_50_ ≤ 2 µM, and SI ≥ 20).

## Supplementary Materials

Figures S1–S29: 1H-NMR, 13C-NMR and HRMS data spectra of compounds 2, 9, 16, 24, 41, 44, 45, 46, 48 and 49.

## References

[1] World Health Organization (WHO) World Malaria Report 2019. https://www.who.int/publications-detail-redirect/world-malaria-report-2019.

[2] Mbengue A., Bhattacharjee S., Pandharkar T., Liu H., Estiu G., Stahelin R.V., Rizk S.S., Njimoh D.L., Ryan Y., Chotivanich K. (2015). Molecular Mechanism of Artemisinin Resistance in *Plasmodium falciparum* Malaria. Nature.

[3] Müller O., Lu G.Y., von Seidlein L. (2019). Geographic Expansion of Artemisinin Resistance. J. Travel Med..

[4] Ocan M., Akena D., Nsobya S., Kamya M.R., Senono R., Kinengyere A.A., Obuku E. (2019). K13-Propeller Gene Polymorphisms in *Plasmodium falciparum* Parasite Population in Malaria Affected Countries: A Systematic Review of Prevalence and Risk Factors. Malar. J..

[5] Kobayashi S., Ueno M., Suzuki R., Ishitani H., Kim H.-S., Wataya Y. (1999). Catalytic Asymmetric Synthesis of Antimalarial Alkaloids Febrifugine and Isofebrifugine and Their Biological Activity. J. Org. Chem..

[6] Gellis A., Kieffer C., Primas N., Lanzada G., Giorgi M., Verhaeghe P., Vanelle P. (2014). A New DMAP-Catalyzed and Microwave-Assisted Approach for Introducing Heteroarylamino Substituents at Position-4 of the Quinazoline Ring. Tetrahedron.

[7] Cohen A., Suzanne P., Lancelot J.-C., Verhaeghe P., Lesnard A., Basmaciyan L., Hutter S., Laget M., Dumètre A., Paloque L. (2015). Discovery of New Thienopyrimidinone Derivatives Displaying Antimalarial Properties toward Both Erythrocytic and Hepatic Stages of Plasmodium. Eur. J. Med. Chem..

[8] Desroches J., Kieffer C., Primas N., Hutter S., Gellis A., El-Kashef H., Rathelot P., Verhaeghe P., Azas N., Vanelle P. (2017). Discovery of New Hit-Molecules Targeting *Plasmodium falciparum* through a Global SAR Study of the 4-Substituted-2-Trichloromethylquinazoline Antiplasmodial Scaffold. Eur. J. Med. Chem..

[9] Verhaeghe P., Azas N., Gasquet M., Hutter S., Ducros C., Laget M., Rault S., Rathelot P., Vanelle P. (2008). Synthesis and Antiplasmodial Activity of New 4-Aryl-2-Trichloromethylquinazolines. Bioorg. Med. Chem. Lett..

[10] Castera-Ducros C., Azas N., Verhaeghe P., Hutter S., Garrigue P., Dumètre A., Mbatchi L., Laget M., Remusat V., Sifredi F. (2011). Targeting the Human Malaria Parasite *Plasmodium falciparum*: In Vitro Identification of a New Antiplasmodial Hit in 4-Phenoxy-2-Trichloromethylquinazoline Series. Eur. J. Med. Chem..

[11] Gellis A., Primas N., Hutter S., Lanzada G., Remusat V., Verhaeghe P., Vanelle P., Azas N. (2016). Looking for New Antiplasmodial Quinazolines: DMAP-Catalyzed Synthesis of 4-Benzyloxy- and 4-Aryloxy-2-Trichloromethylquinazolines and Their in Vitro Evaluation toward *Plasmodium falciparum*. Eur. J. Med. Chem..

[12] Primas N., Verhaeghe P., Cohen A., Kieffer C., Dumètre A., Hutter S., Rault S., Rathelot P., Azas N., Vanelle P. (2012). A New Synthetic Route to Original Sulfonamide Derivatives in 2-Trichloromethylquinazoline Series: A Structure-Activity Relationship Study of Antiplasmodial Activity. Molecules.

[13] Verhaeghe P., Rathelot P., Gellis A., Rault S., Vanelle P. (2006). Highly Efficient Microwave Assisted α-Trichlorination Reaction of α-Methylated Nitrogen Containing Heterocycles. Tetrahedron.

[14] Verhaeghe P., Azas N., Hutter S., Castera-Ducros C., Laget M., Dumètre A., Gasquet M., Reboul J.-P., Rault S., Rathelot P. (2009). Synthesis and in Vitro Antiplasmodial Evaluation of 4-Anilino-2-Trichloromethylquinazolines. Bioorg. Med. Chem..

[15] Verhaeghe P., Dumètre A., Castera-Ducros C., Hutter S., Laget M., Fersing C., Prieri M., Yzombard J., Sifredi F., Rault S. (2011). 4-Thiophenoxy-2-Trichloromethyquinazolines Display in Vitro Selective Antiplasmodial Activity against the Human Malaria Parasite *Plasmodium falciparum*. Bioorg. Med. Chem. Lett..

[16] Stumpfe D., Bajorath J. (2012). Exploring Activity Cliffs in Medicinal Chemistry. J. Med. Chem..

[17] Caballero-García G., Romero-Ortega M., Barroso-Flores J. (2016). Reactivity of Electrophilic Chlorine Atoms Due to σ-Holes: A Mechanistic Assessment of the Chemical Reduction of a Trichloromethyl Group by Sulfur Nucleophiles. Phys. Chem. Chem. Phys..

[18] Rosenthal P.J. (2020). Falcipain Cysteine Proteases of Malaria Parasites: An Update. Biochim. Biophys. Acta Proteins Proteom..

[19] Mosmann T. (1983). Rapid Colorimetric Assay for Cellular Growth and Survival: Application to Proliferation and Cytotoxicity Assays. J. Immunol. Methods.

[20] Trager W., Jensen J.B. (1976). Human Malaria Parasites in Continuous Culture. Science.

[21] Guiguemde W.A., Shelat A.A., Bouck D., Duffy S., Crowther G.J., Davis P.H., Smithson D.C., Connelly M., Clark J., Zhu F. (2010). Chemical Genetics of *Plasmodium falciparum*. Nature.

