# Supplementary material for: Synthesis and Antiplasmodial Evaluation of 4-Carboxamido- and 4-Alkoxy-2-Trichloromethyl Quinazolines
Source: Molecules. 2020 Aug 27;25(17):3929. doi: 10.3390/molecules25173929 (PMC7504092; doi:10.3390/molecules25173929)

Supplementary Materials

Synthesis and Antiplasmodial Evaluation of 4-Carboxamido- and 4-Alkoxy-2-trichloromethyl quinazolines.

Dyhia Amrane[1†], Armand Gellis[1†], Sébastien Hutter[2], Marion Prieri[1], Pierre Verhaeghe[3], Nadine Azas[2], Patrice Vanelle[1*] and Nicolas Primas[1*]

[1]Aix Marseille Univ, CNRS, ICR UMR 7273, Equipe Pharmaco-Chimie Radicalaire, Faculté de Pharmacie, 27 Boulevard Jean Moulin – CS30064, 13385 Marseille cedex 05, France.

[2]Aix Marseille Univ, IHU Méditerranée Infection, UMR VITROME, IRD, SSA, Mycology & Tropical Eucaryotic pathogens, 19-21 Boulevard Jean Moulin, 13005 Marseille cedex 05, France.

[3]LCC-CNRS Université de Toulouse, CNRS, UPS, Toulouse, France

†Co-first author, these authors contributed equally to this work.

TABLE OF CONTENT

Figure S1. ^{1}H-NMR spectra of (2) ... 3

Figure S2. ^{13}C-NMR spectra of (2) ... 3

Figure S3. HRMS spectra of (2) ... 4

Figure S4. ^{1}H-NMR spectra of (9) ... 5

Figure S5. ^{13}C-NMR spectra of (9) ... 5

Figure S6. HRMS spectra of (9) ... 6

Figure S7. ^{1}H-NMR spectra of (16) ... 7

Figure S8. ^{13}C-NMR spectra of (16) ... 7

Figure S9. HRMS spectra of (16) ... 8

Figure S10. ^{1}H-NMR spectra of (24) ... 9

Figure S11. ^{13}C-NMR spectra of (24) ... 9

Figure S12. HRMS spectra of (24) ... 10

Figure S13. ^{1}H-NMR spectra of (41) ... 11

Figure S14. ^{13}C-NMR spectra of (41) ... 11

Figure S15. ^{1}H-NMR spectra of (44) ... 12

Figure S16. ^{13}C-NMR spectra of (44) ... 12

Figure S17. HRMS spectra of (44) ... 13

Figure S18. ^{1}H-NMR spectra of (45) 14
Figure S19. ^{13}C-NMR spectra of (45) 14
Figure S20. HRMS spectra of (45) 15
Figure S21. ^{1}H-NMR spectra of (46) 16
Figure S22. ^{13}C-NMR spectra of (46) 16
Figure S23. HRMS spectra of (46) 17
Figure S24. ^{1}H-NMR spectra of (48) 18
Figure S25. ^{13}C-NMR spectra of (48) 18
Figure S26. HRMS spectra of (48) 19
Figure S27. ^{1}H-NMR spectra of (49) 20
Figure S28. ^{13}C-NMR spectra of (49) 20
Figure S29. HRMS spectra of (48) 21

Figure S1. ^{1}H-NMR spectra of (2)

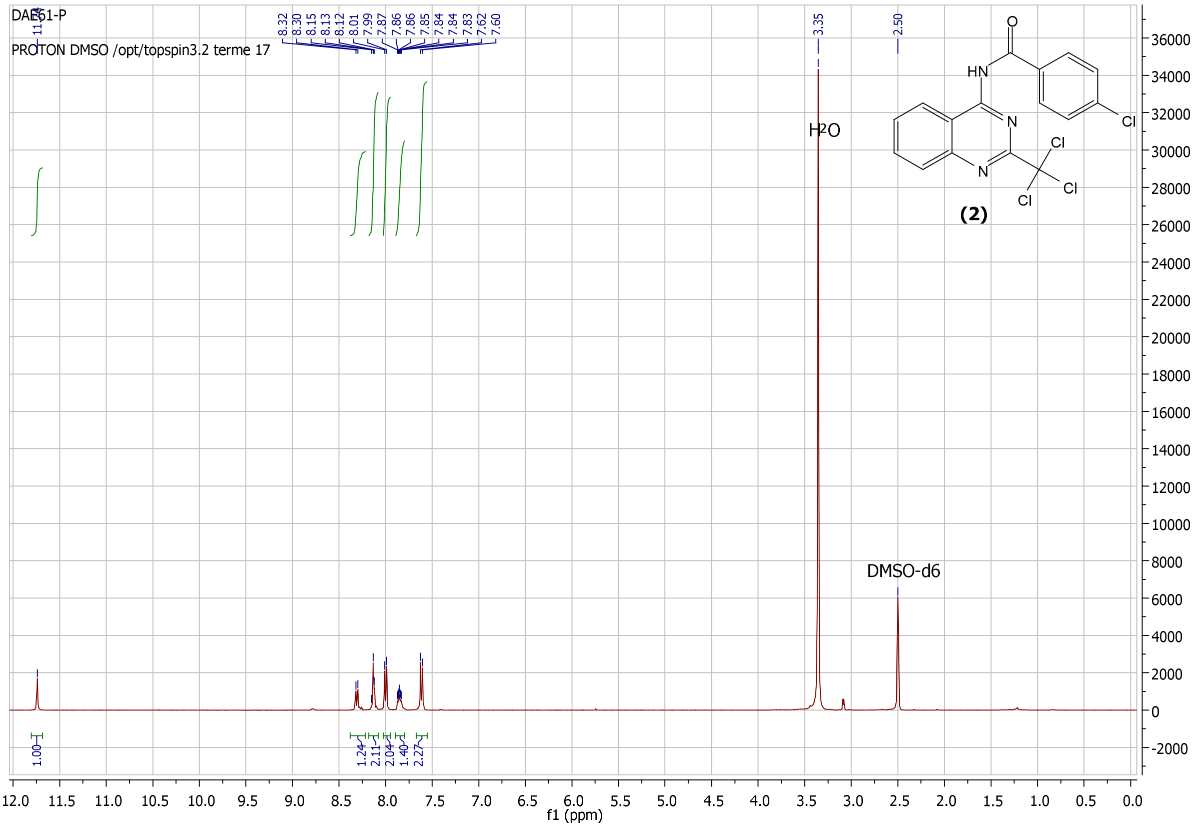

Figure S2. ^{13}C-NMR spectra of (2)

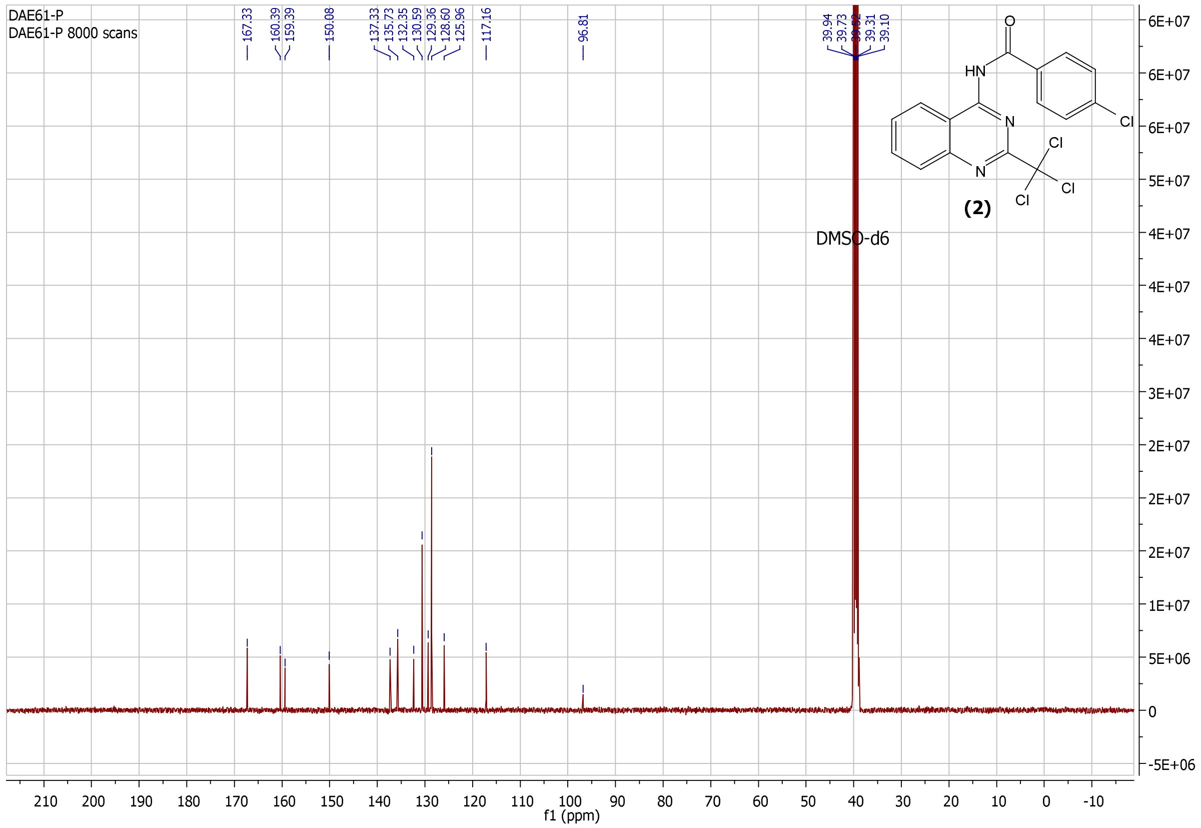

Figure S3. HRMS spectra of (2)

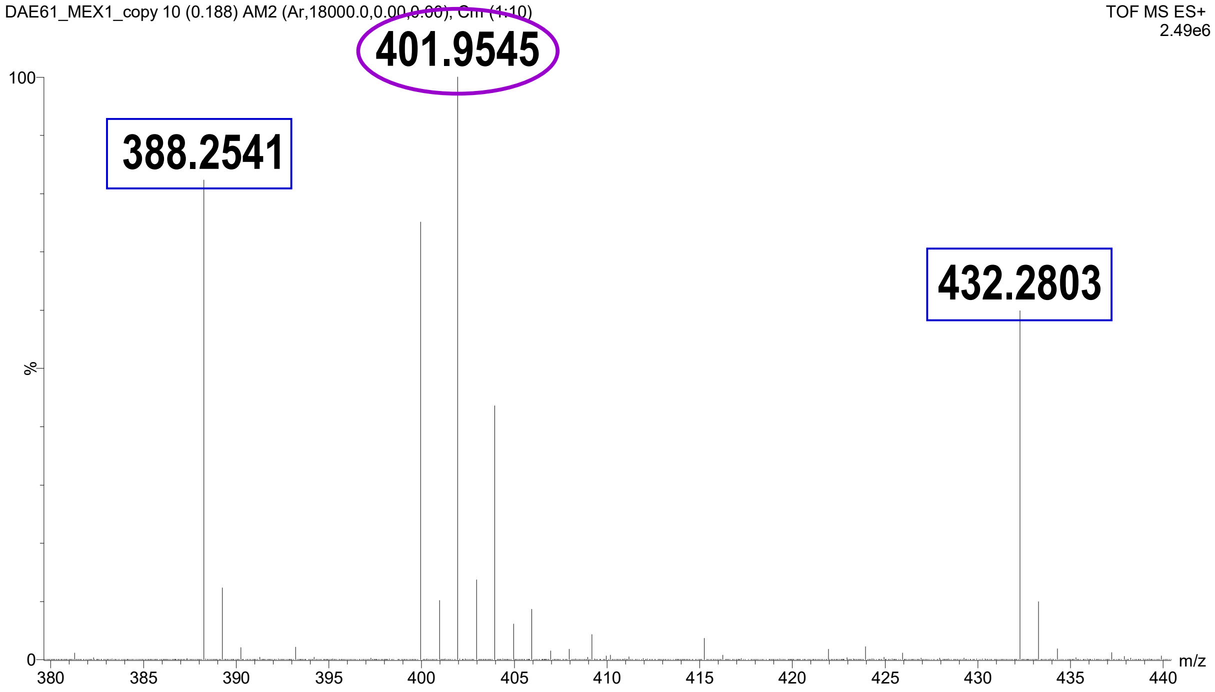

Figure S4. ^{1}H-NMR spectra of (9)

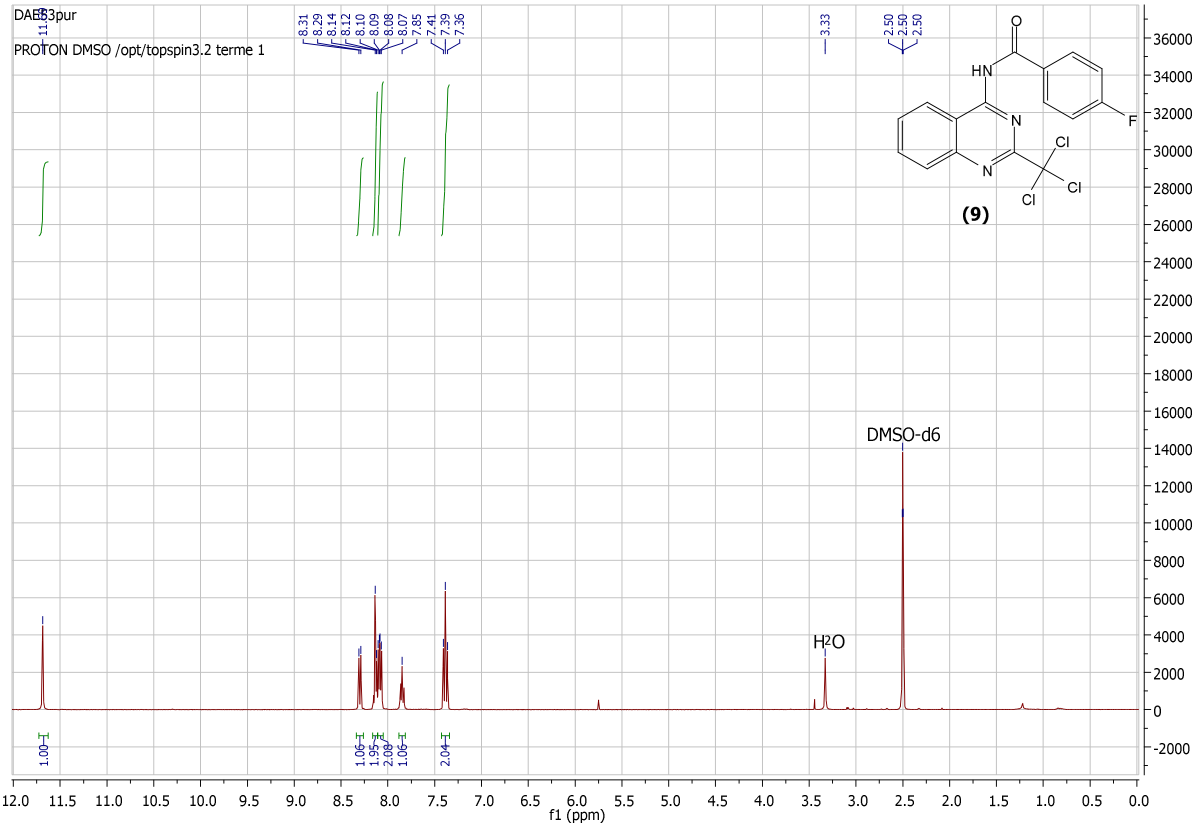

Figure S5. ^{13}C-NMR spectra of (9)

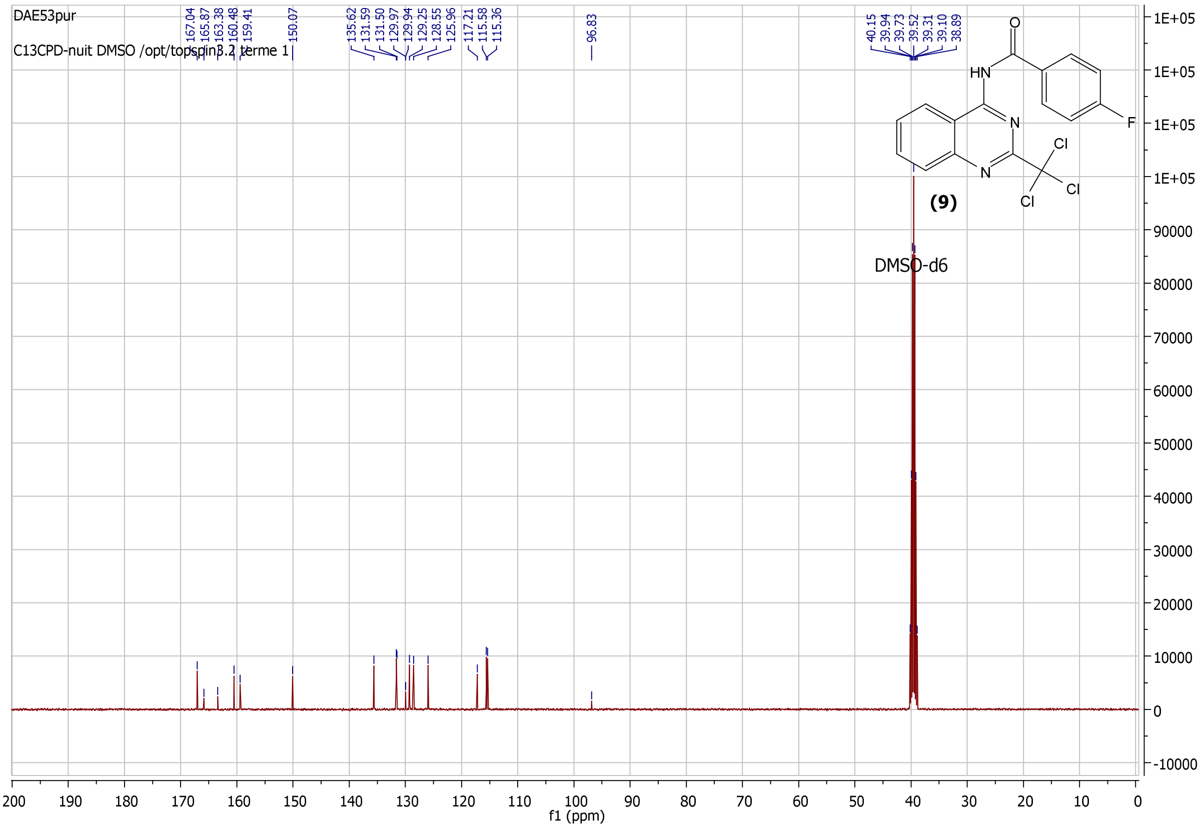

Figure S6. HRMS spectra of (9)

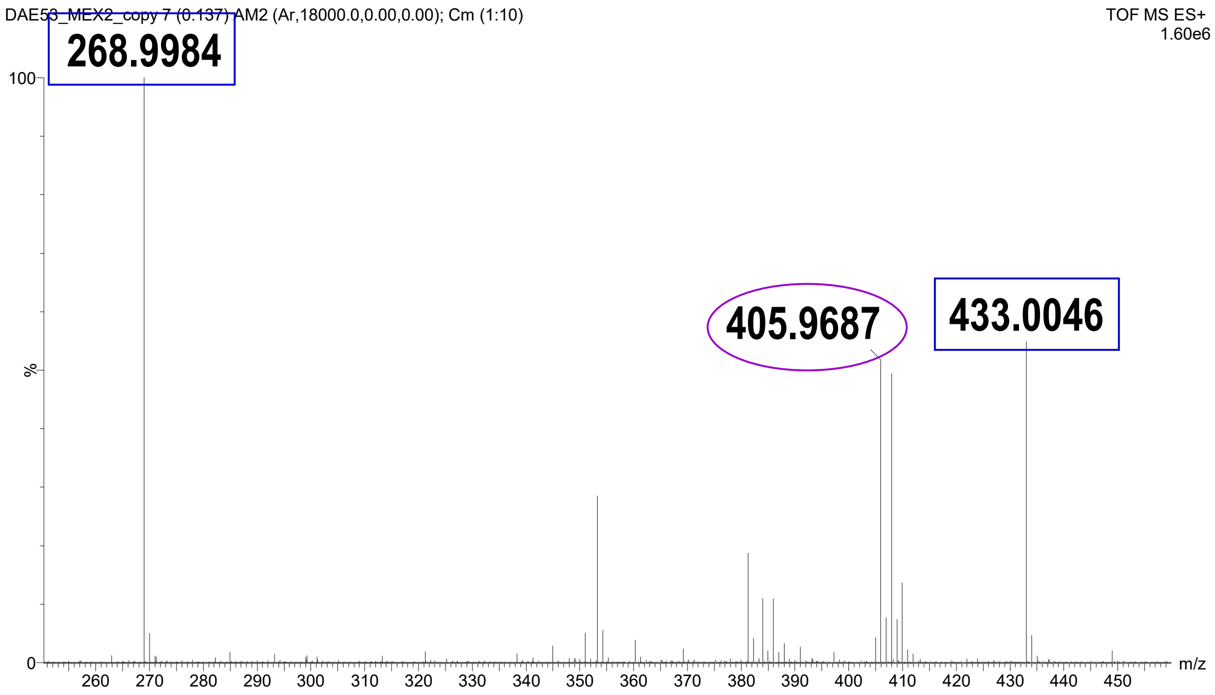

Figure S7. ¹H-NMR spectra of (16)

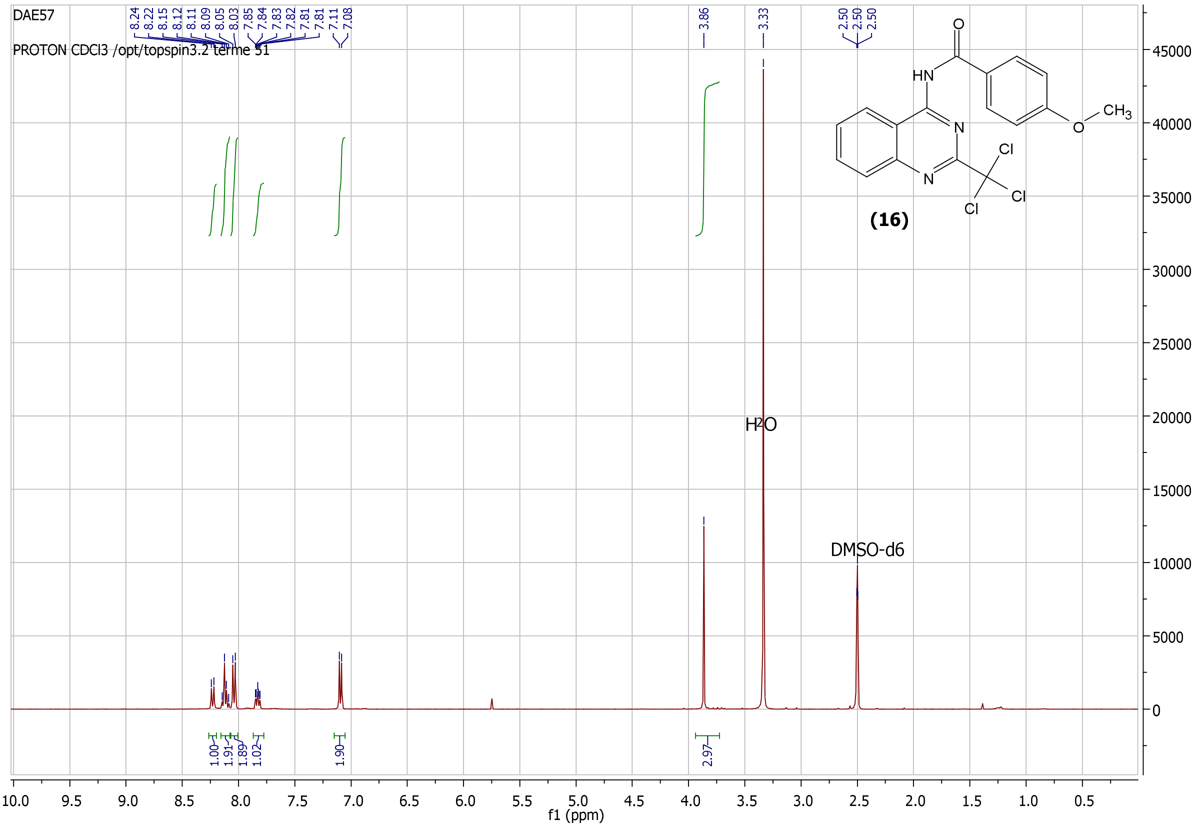

Figure S8. ¹³C-NMR spectra of (16)

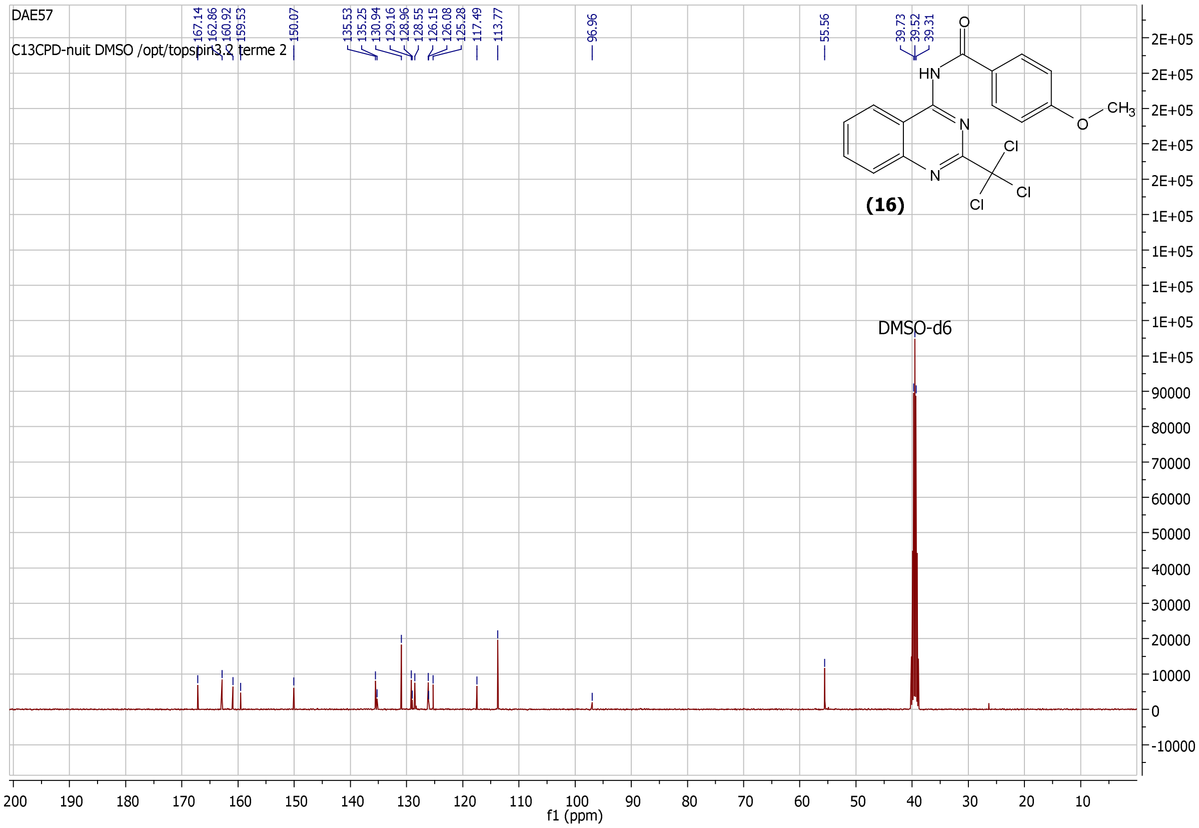

Figure S9. HRMS spectra of (16)

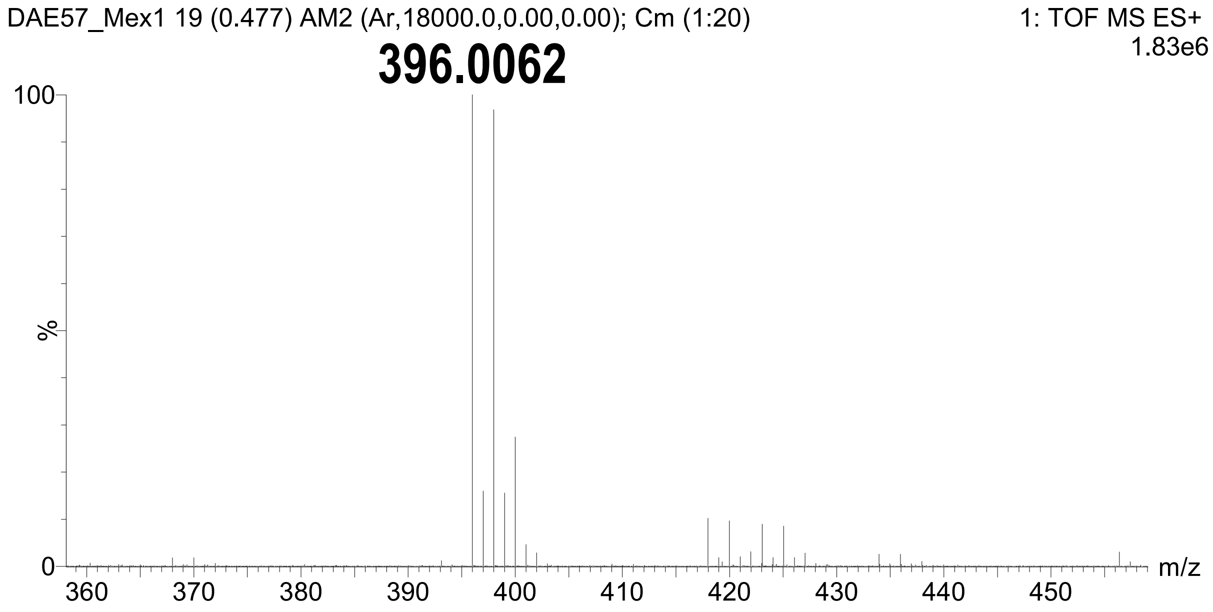

Figure S10. **^{1}H-NMR spectra of (24)**

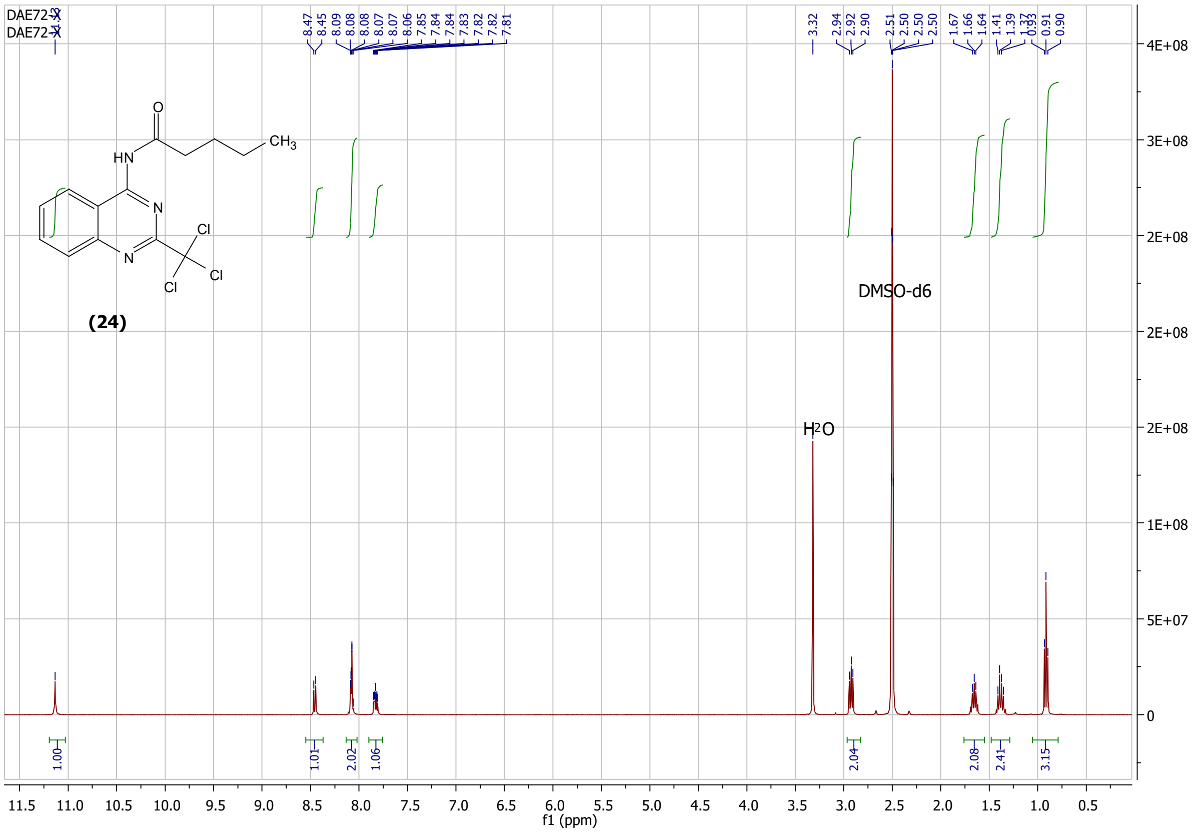

Figure S11. **^{13}C-NMR spectra of (24)**

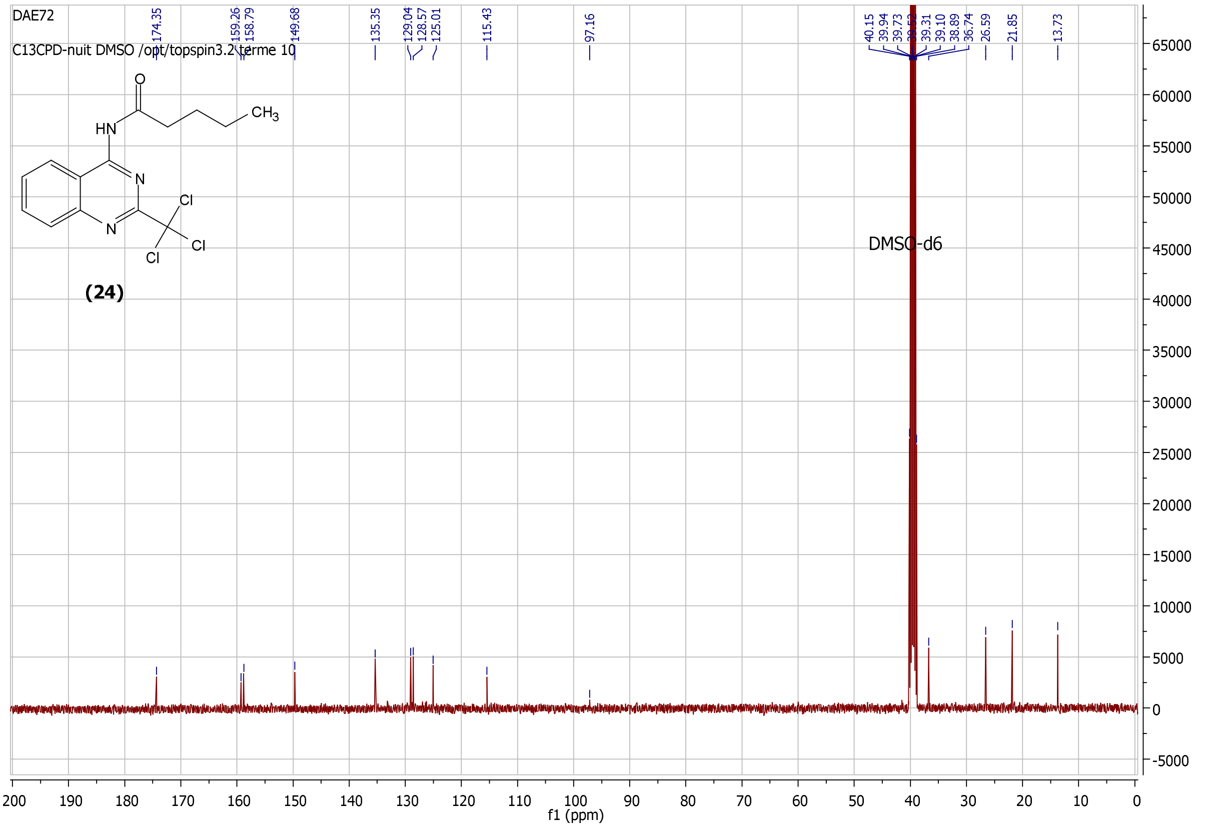

Figure S12. HRMS spectra of (24)

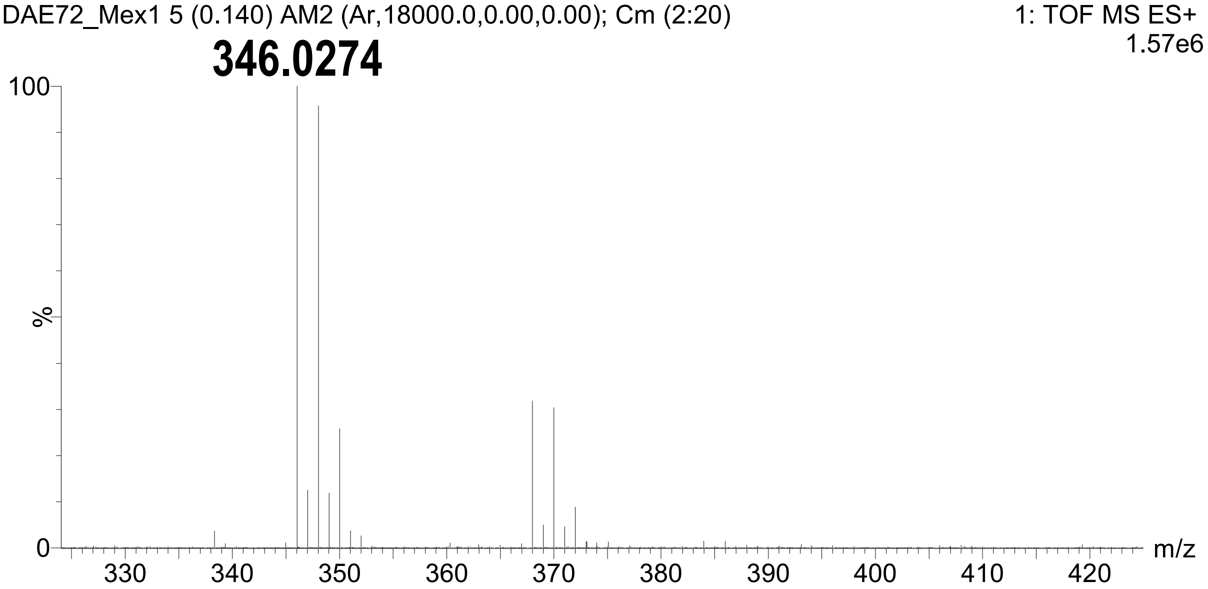

Figure S13. ¹H-NMR spectra of (41)

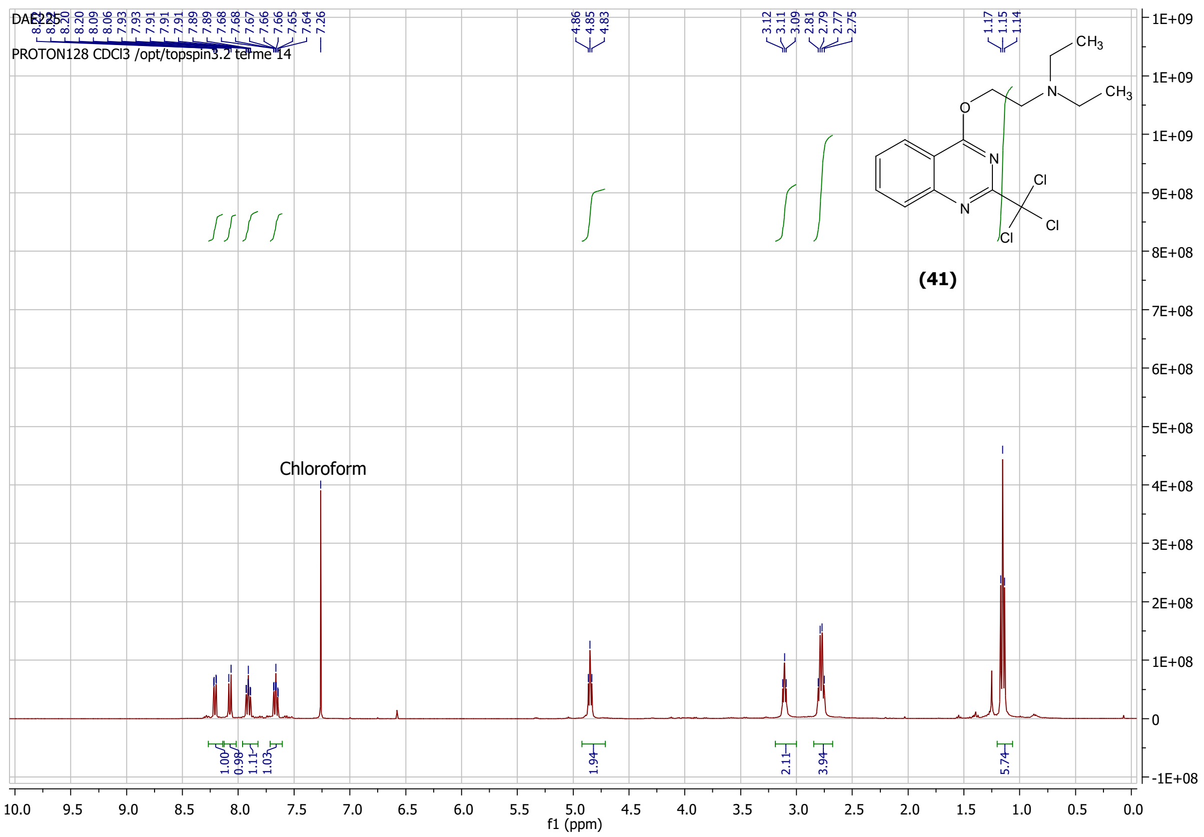

Figure S14. ¹³C-NMR spectra of (41)

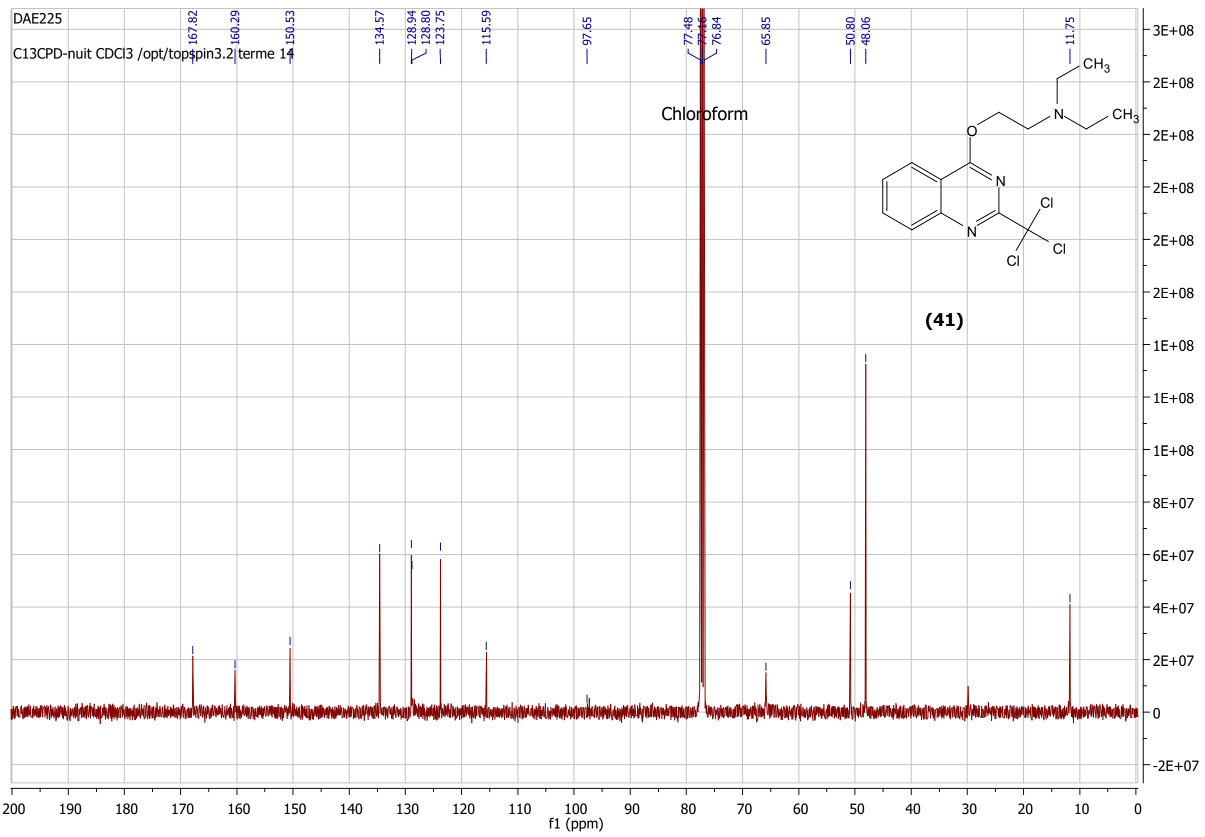

Figure S15. ^{1}H-NMR spectra of (44)

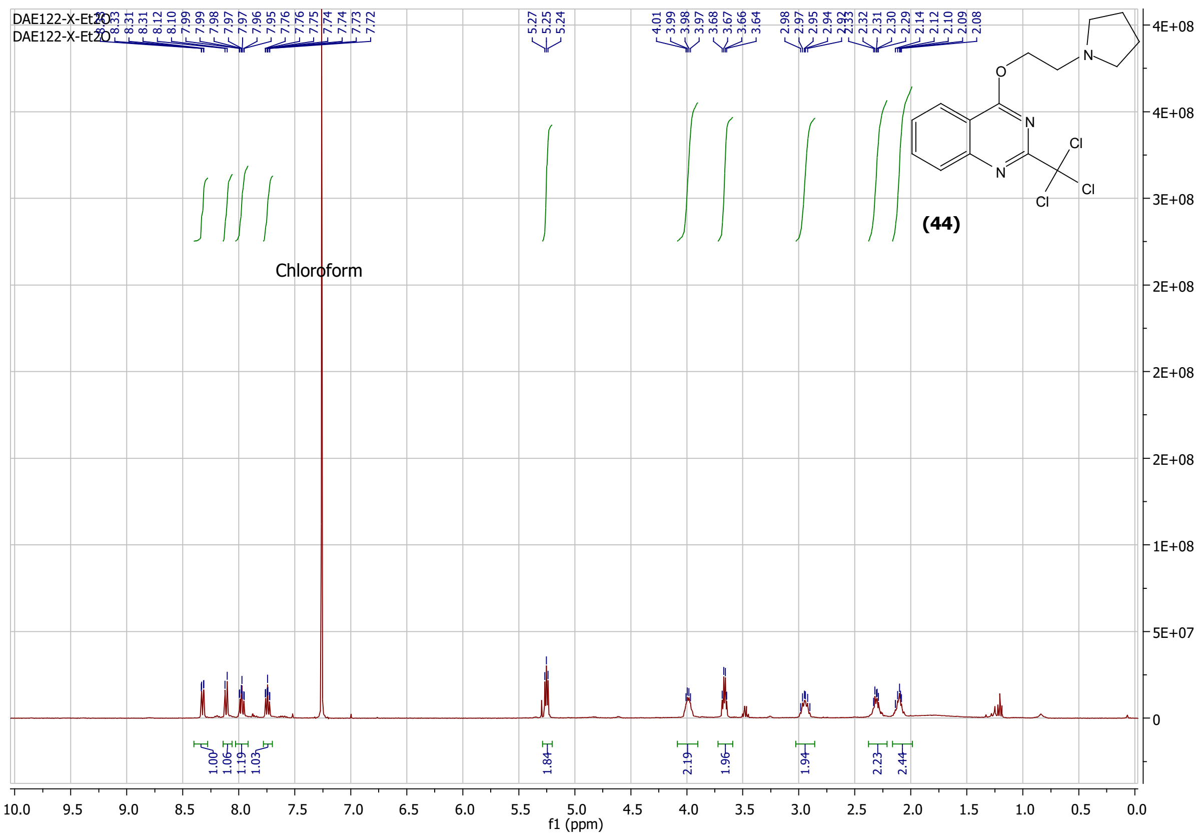

Figure S16. ^{13}C-NMR spectra of (44)

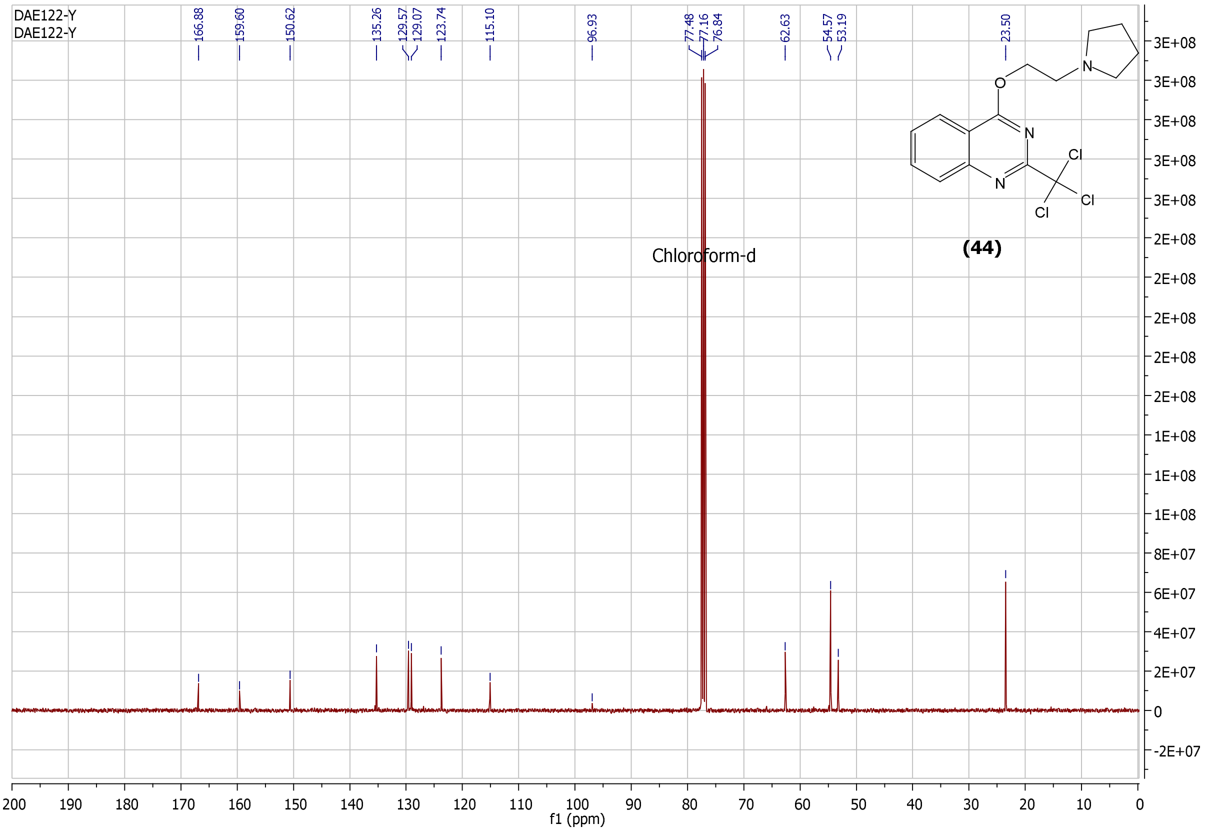

Figure S17. HRMS spectra of (44)

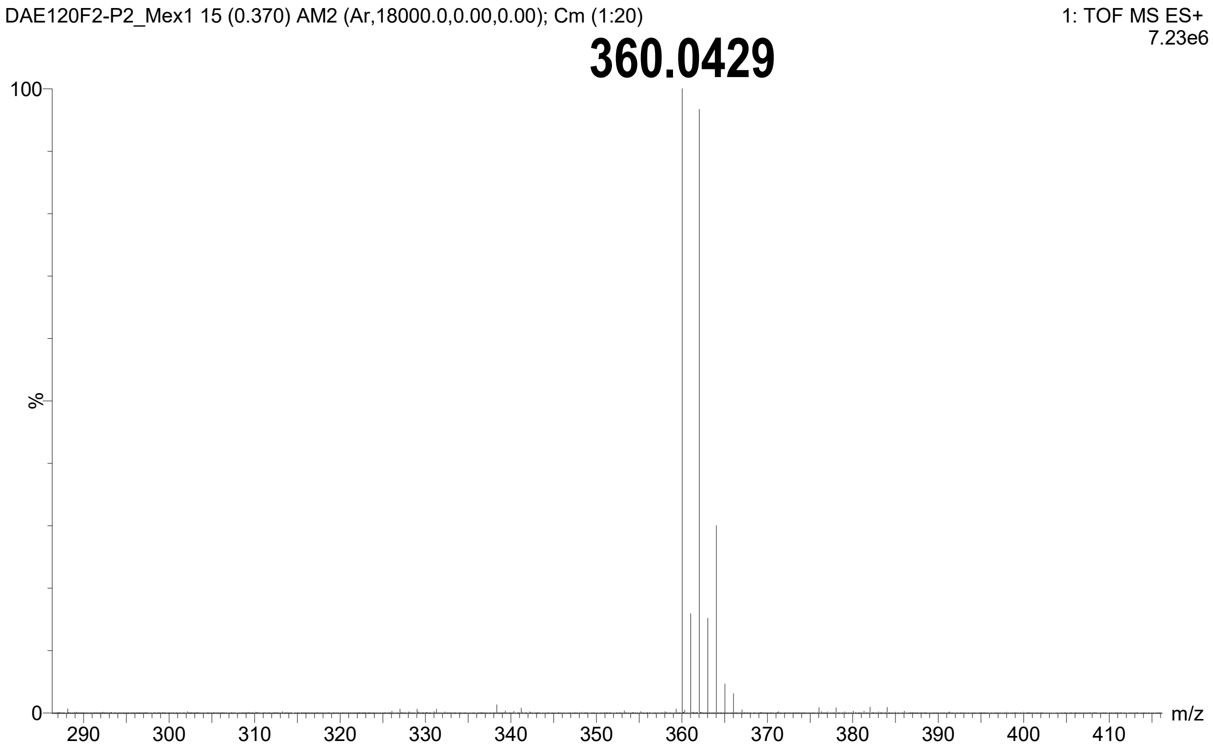

Figure S18. ^{1}H-NMR spectra of (45)

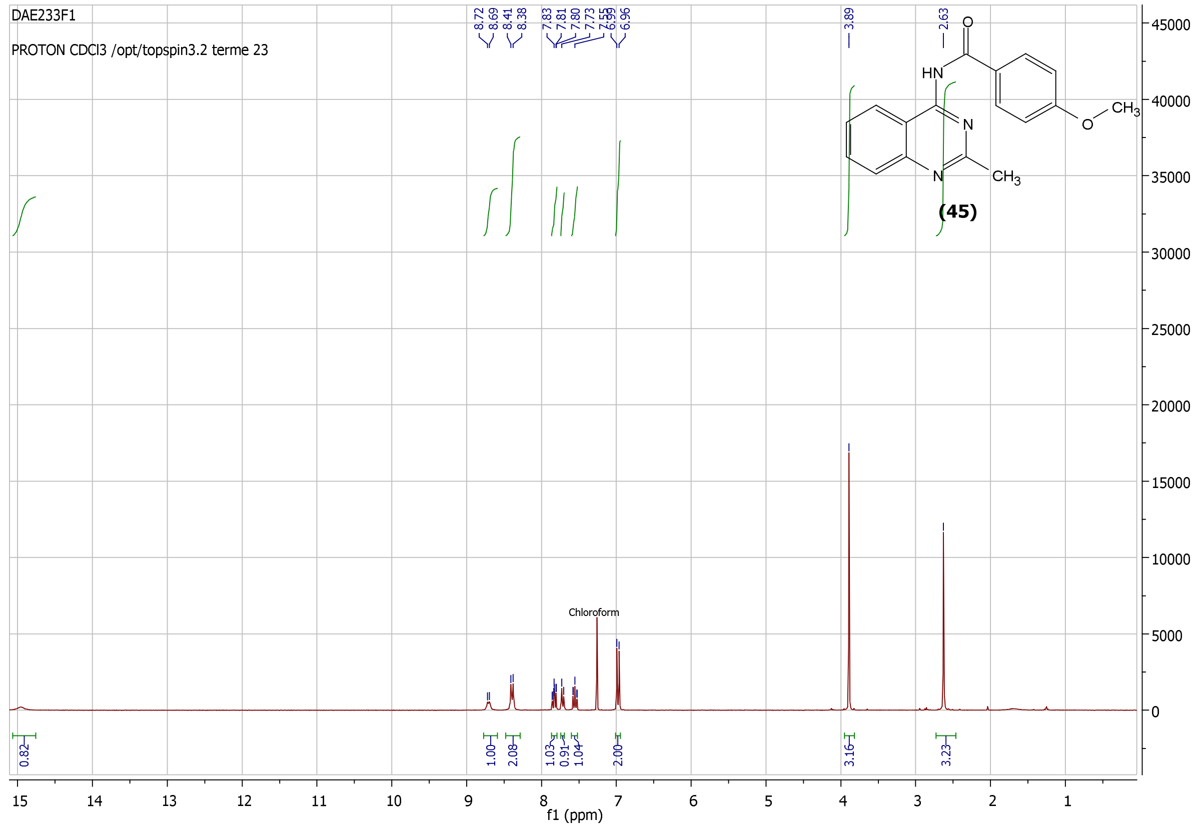

Figure S19. ^{13}C-NMR spectra of (45)

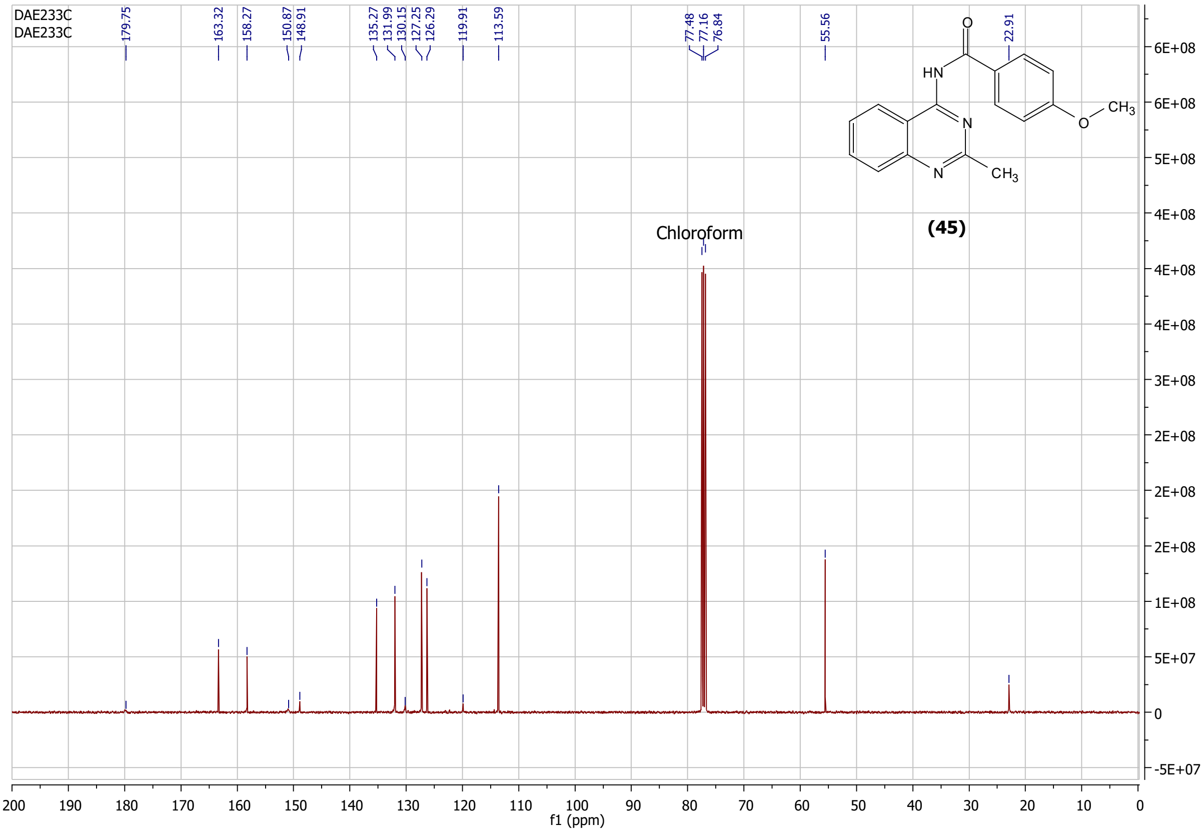

Figure S20. HRMS spectra of (45)

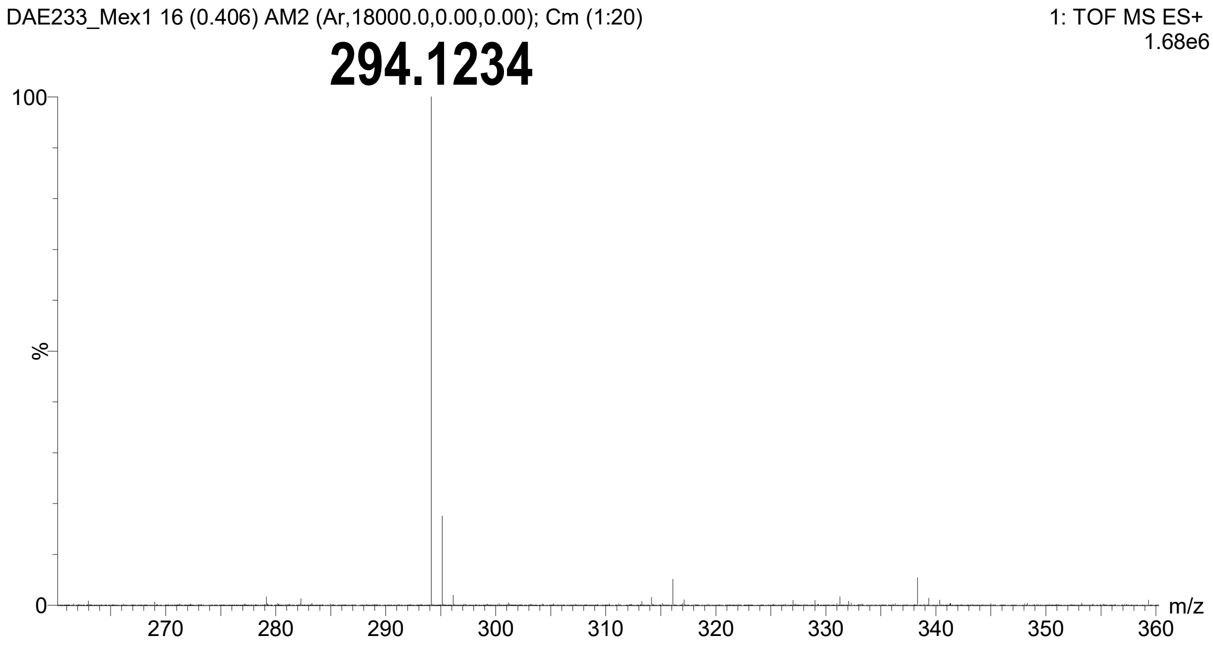

Figure S21. ^{1}H-NMR spectra of (46)

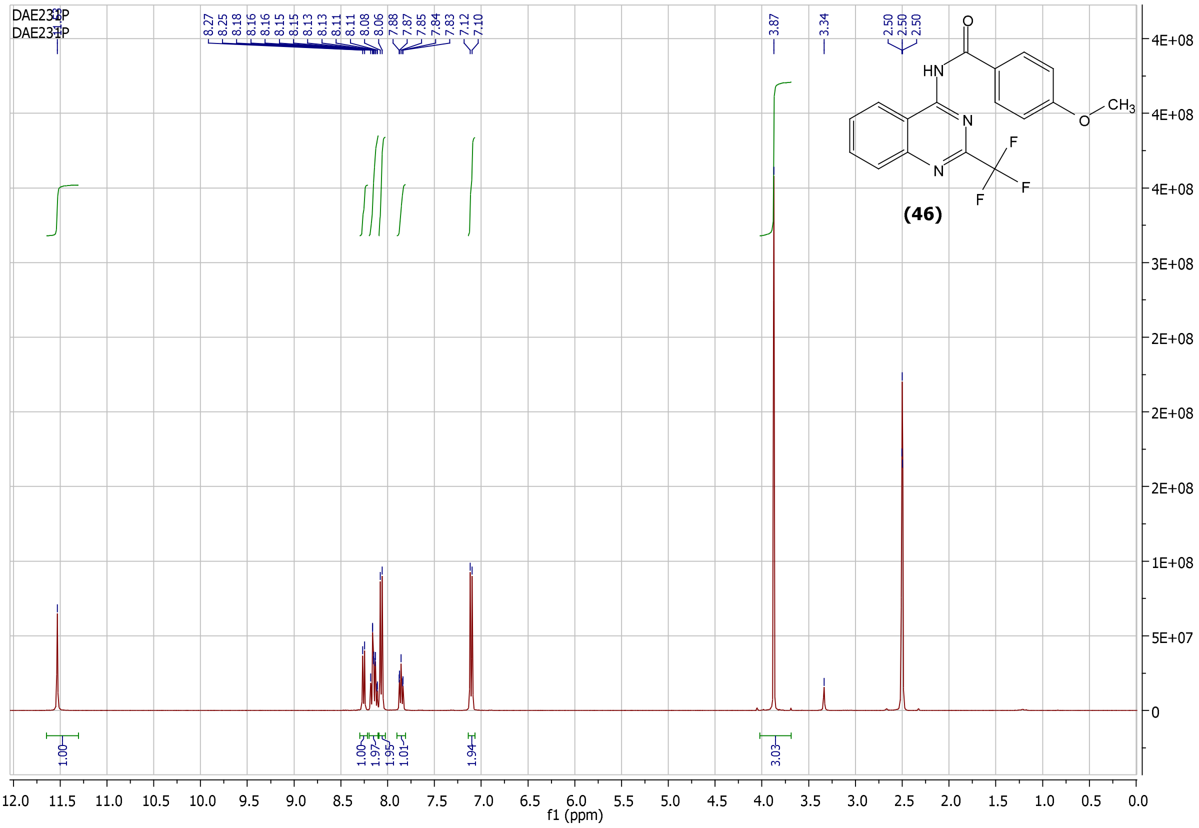

Figure S22. ^{13}C-NMR spectra of (46)

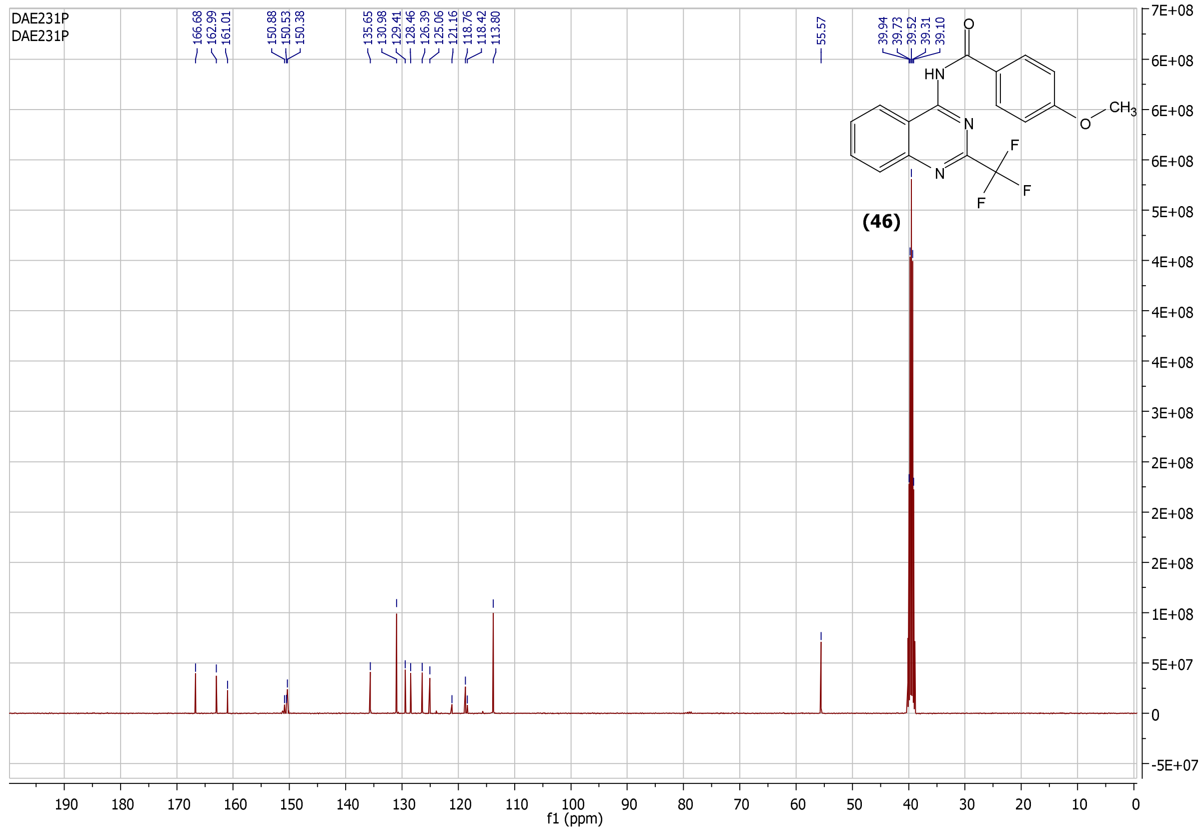

Figure S23. HRMS spectra of (46)

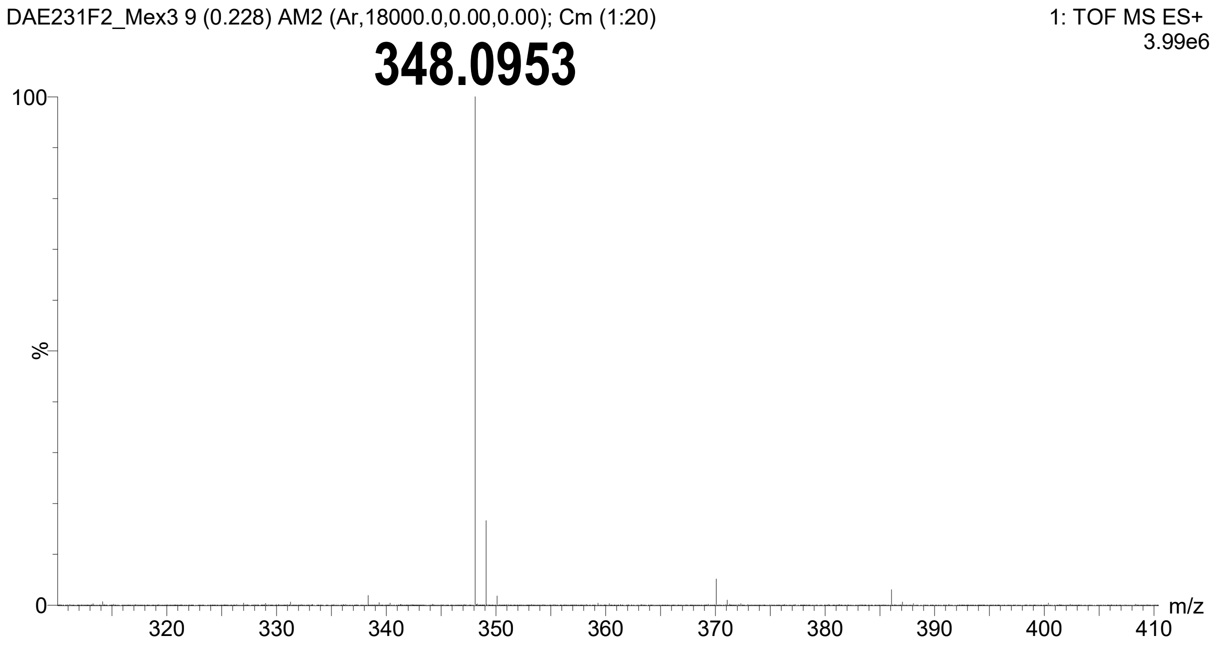

Figure S24. ^{1}H-NMR spectra of (48)

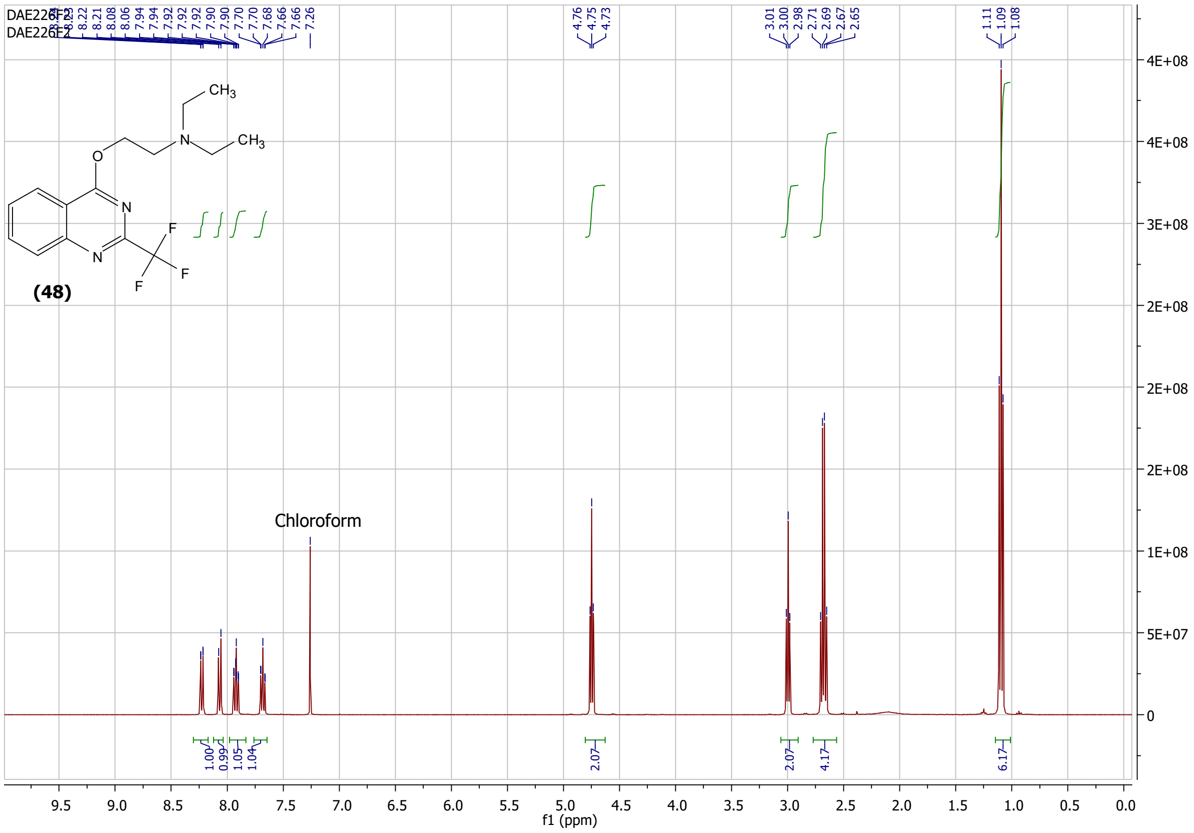

Figure S25. ^{13}C-NMR spectra of (48)

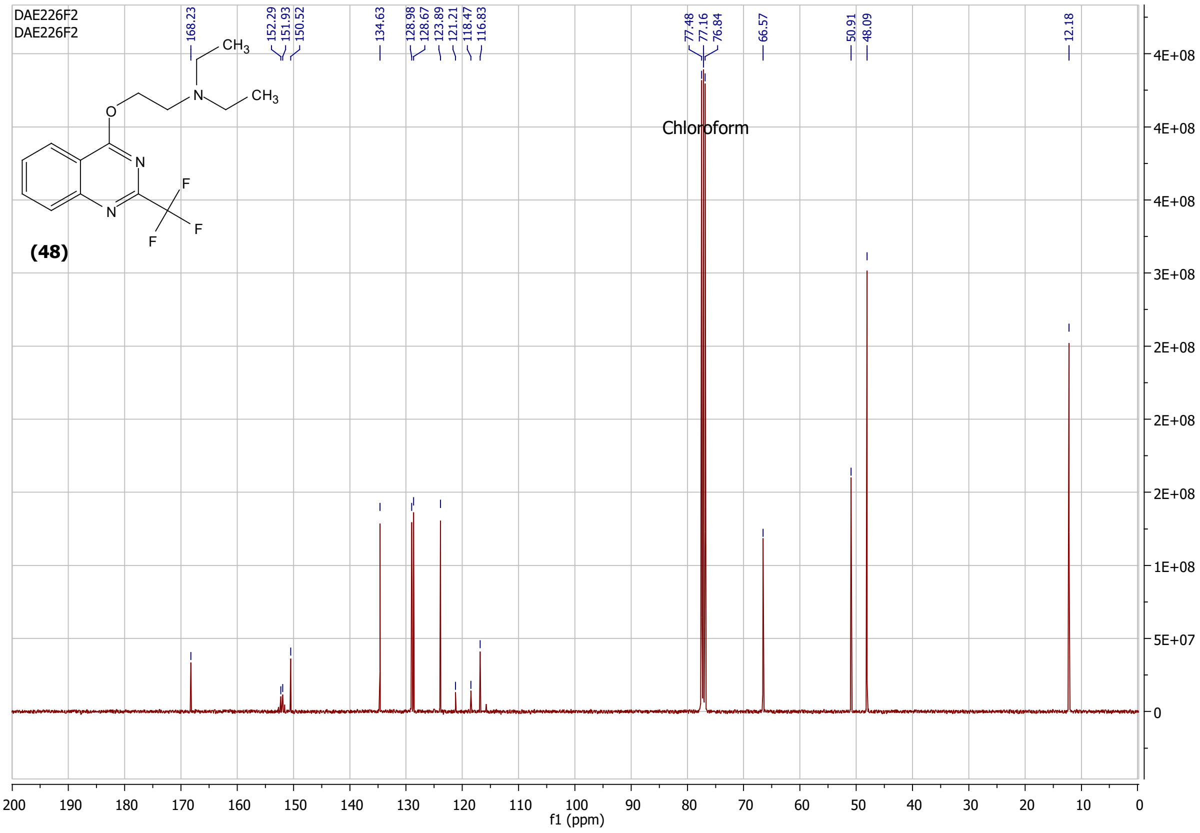

Figure S26. HRMS spectra of (48)

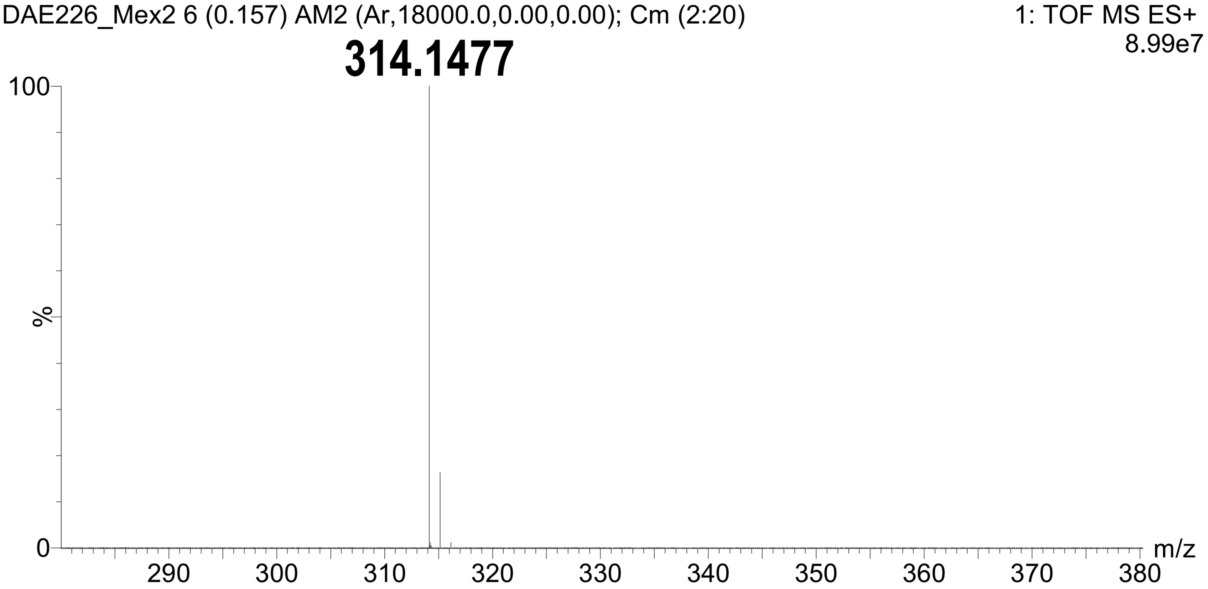

Figure S27. ^{1}H-NMR spectra of (49)

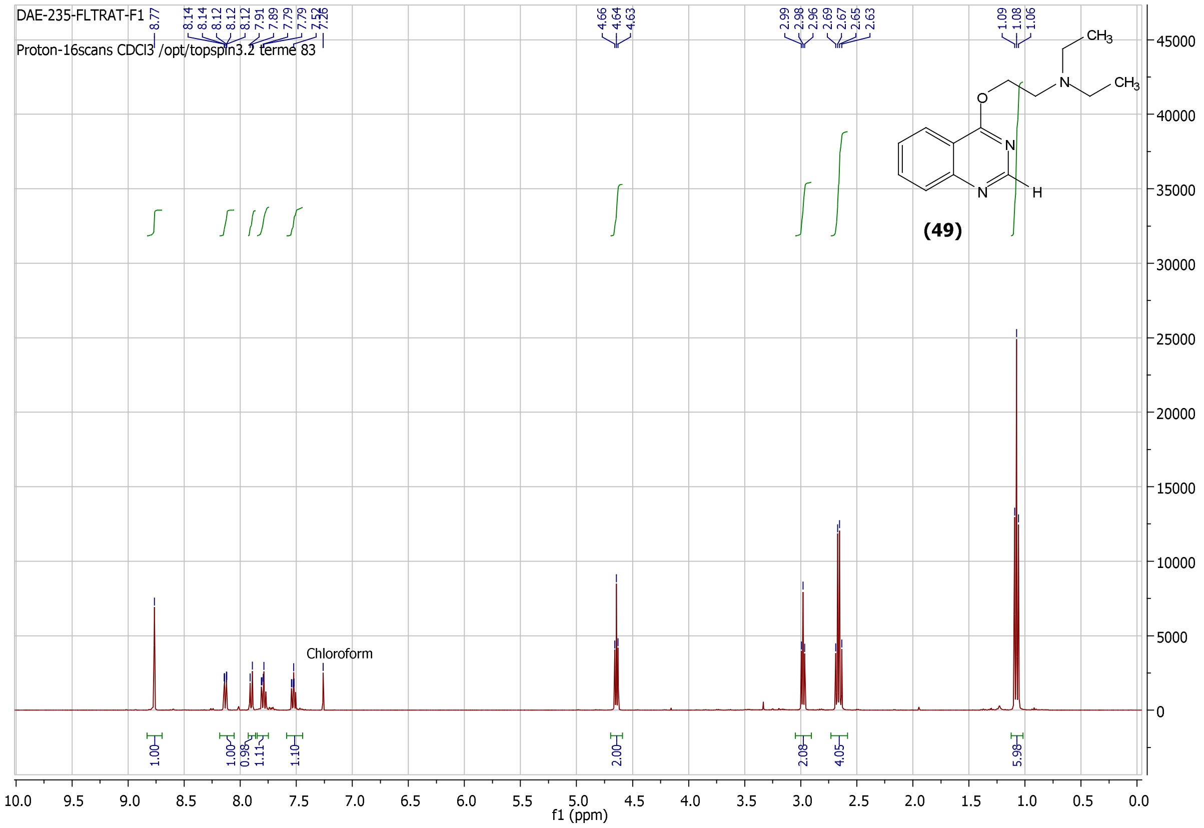

Figure S28. ^{13}C-NMR spectra of (49)

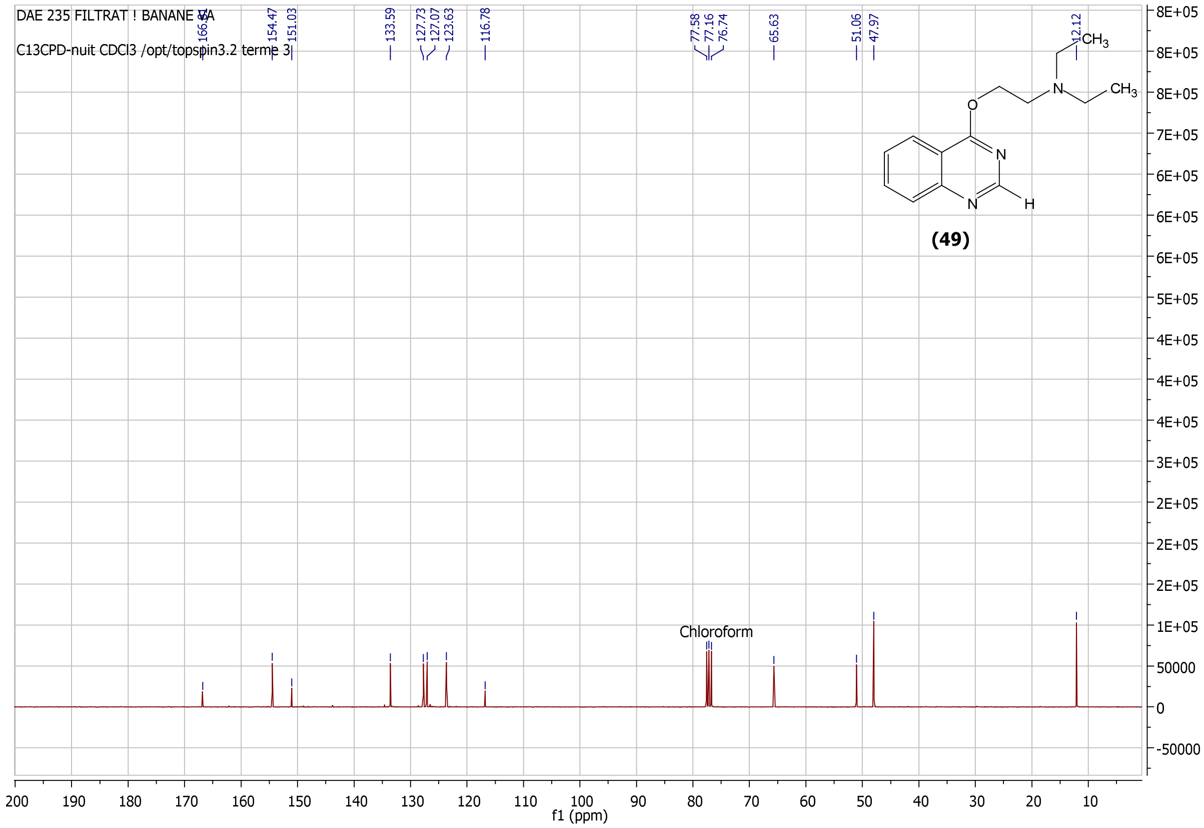

Figure S29. HRMS spectra of (48)

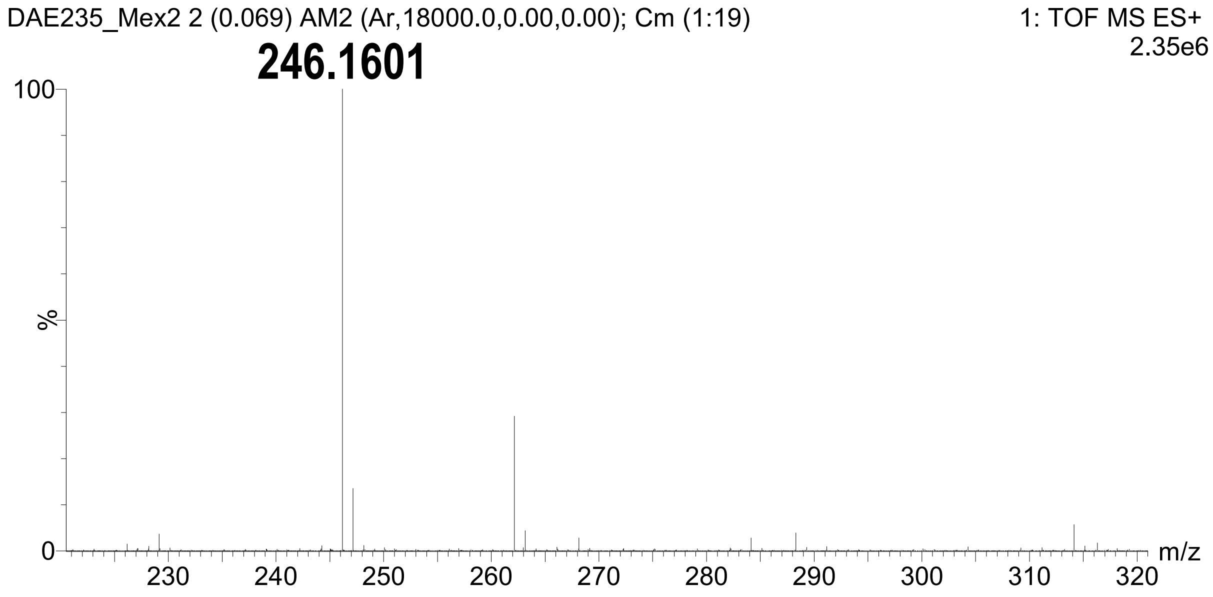

Supplement: Supplementary file 1 [file molecules-25-03929-s001.pdf]
